# The importance of imperfect pre-clinical models in adolescent idiopathic scoliosis

**DOI:** 10.1242/dmm.052438

**Published:** 2025-09-01

**Authors:** Diane S. Sepich, Ryan S. Gray, Nadav Ahituv, Christina A. Gurnett, Jonathan J. Rios, Lila Solnica-Krezel, Carol A. Wise

**Affiliations:** ^1^ Department of Developmental Biology, Washington University School of Medicine, St. Louis, MO 63110, USA; ^2^ Department of Pediatrics, Dell Pediatric Research Institute, University of Texas at Austin Dell Medical School, Austin, TX 78723, USA; ^3^ Department of Bioengineering and Therapeutic Sciences, University of California San Francisco, San Francisco, CA 94158, USA; ^4^ Institute for Human Genetics, University of California San Francisco, San Francisco, CA 94158, USA; ^5^ Department of Neurology, Washington University School of Medicine, St. Louis, MO 63110, USA; ^6^ Department of Orthopaedic Surgery, University of Texas Southwestern Medical Center, Dallas, TX 75235, USA; ^7^ McDermott Center for Human Growth and Development, University of Texas Southwestern Medical Center, Dallas, TX 75235, USA; ^8^ Department of Pediatrics, University of Texas Southwestern Medical Center, Dallas, TX 75235, USA; ^9^ Center for Translational Research, Scottish Rite for Children, 2222 Welborn St., Dallas, TX 75219, USA

## Abstract

Adolescent idiopathic scoliosis (AIS) is a twisting spinal deformity that occurs in otherwise healthy children at the time of rapid pre-pubescent growth. AIS affects ∼3% of children worldwide and is the most common musculoskeletal diagnosis in pediatric populations, posing a significant physiological, psychosocial and financial burden to patients. Genetic predisposition is a clear and major contributor to AIS, and insights from genomic discoveries are inspiring translational studies ultimately aimed at developing novel diagnostics and therapies. Pre-clinical animal models of AIS are now essential to validate human genetic findings, understand gene-by-environment interactions, and speed etiologic and therapeutic discovery. In this Perspective, we highlight the current status of pre-clinical models of AIS and discuss the challenges posed by the nature of the disorder combined with the limitations of standard approaches. Current research suggests that straightforward genetic targeting of orthologous AIS disease genes in vertebrates may not necessarily yield equivalent physiological phenotypes but nevertheless can be utilized to understand disease mechanisms. Longer-term, appropriately complex models are needed to fully recapitulate the human AIS phenotype arising from genetic, physiological and mechanical interactions.

## Teaser

**Summary:** Pre-clinical models of adolescent idiopathic scoliosis are urgently needed to speed etiologic and therapeutic discoveries, even if they do not perfectly recapitulate all aspects of the disease.

## Introduction

Scoliosis is a term describing a C- or S-shaped deformity of the spine that can be observed in the front-to-back (coronal) plane. Scoliosis in children is typically diagnosed as one of four distinct types: congenital, neuromuscular, syndromic or idiopathic. Congenital scoliosis is caused by malformations of the vertebrae, observable by X-rays, that produce bends in the spine. Neuromuscular scoliosis is secondary to weakness in the muscles of the spine, and syndromic scoliosis is an associated feature of various rare genetic disorders. Most scoliosis is idiopathic, defined as occurring in an otherwise healthy child without vertebral malformations. Idiopathic scoliosis affects ∼3% of the pediatric population worldwide, usually occurring with the pre-pubertal period of rapid growth (Richards et al., 2020; Chan et al., 2002; Hresko, 2013; Murphy and Mooney, 2016). Progressive adolescent idiopathic scoliosis (AIS) requires intervention by bracing or surgery to prevent severe, life-long deformity and associated problems such as pain or lung restriction (Asher and Burton, 2006; Haller et al., 2016; Richards et al., 2020; Hresko, 2013; Weinstein et al., 2008).

An emerging hypothesis to explain AIS is one in which the disease arises from the combined effects of genetic variation, hormonal fluxes and mechanical stresses of rapid adolescent growth

**Fig. 1.**
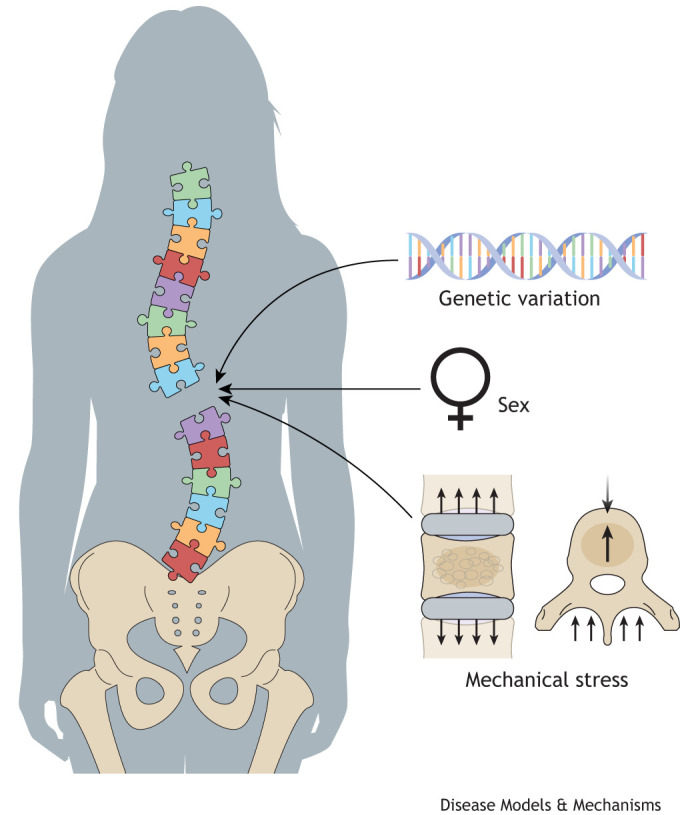
**Adolescent idiopathic scoliosis (AIS) is a spine growth disorder caused by interplay (depicted as puzzle pieces) of the effects of genetics, sex hormones and mechanical stress on the spine during rapid growth.** Missing pieces represent our current lack of understanding of AIS etiology.

Female sex, adolescent growth and genetic factors are clear risk factors for AIS (Karol et al., 1993; Little et al., 2000). Girls have more than fivefold greater risk than boys of developing progressive AIS (Wise et al., 2020). Recurrence risks are elevated threefold and sevenfold for male and female siblings of AIS patients, respectively. Overall heritability estimates vary, ranging from 29% to 88%, with an average of ∼57% (Cheng et al., 2022; Tang et al., 2012, 2021). Accordingly, population-based genome wide association studies (GWAS) have identified a number of AIS susceptibility loci, mostly within non-coding regions of the genome (Khanshour et al., 2018; Kou et al., 2013, 2019; Sharma et al., 2015; Takahashi et al., 2011; Yu et al., 2024). Other studies have linked AIS with rare, protein-altering variations (Baschal et al., 2014; Einarsdottir et al., 2017; Haller et al., 2016, 2018; Patten et al., 2015). An emerging hypothesis to explain AIS is one in which the disease arises from the combined effects of genetic variation, hormonal fluxes and mechanical stresses of rapid adolescent growth (Castelein et al., 2020; Wise et al., 2020) (Fig. 1).

Pre-clinical models are now essential to validate human genetic findings, and to speed etiologic and therapeutic discovery for AIS. Moreover, such models will enable research to address the complex interplay of genetics, chromosomal sex and biomechanical input into the disease. Modern gene-targeting methods, deep sequencing and the increasing understanding of conserved non-coding DNA are empowering the development of new vertebrate models of AIS. Large forward genetic screens in mouse and zebrafish are also yielding mutants that inform understanding of spine development. We argue here that pre-clinical models are now critical in the goal to understand the molecular and environmental underpinnings of AIS. Further, we posit that even models with spine deformities that deviate from the classic ‘idiopathic-like’ phenotype observed in humans could prove valuable to AIS translational research.

## Genetically engineered models of AIS

**Fig. 2.**
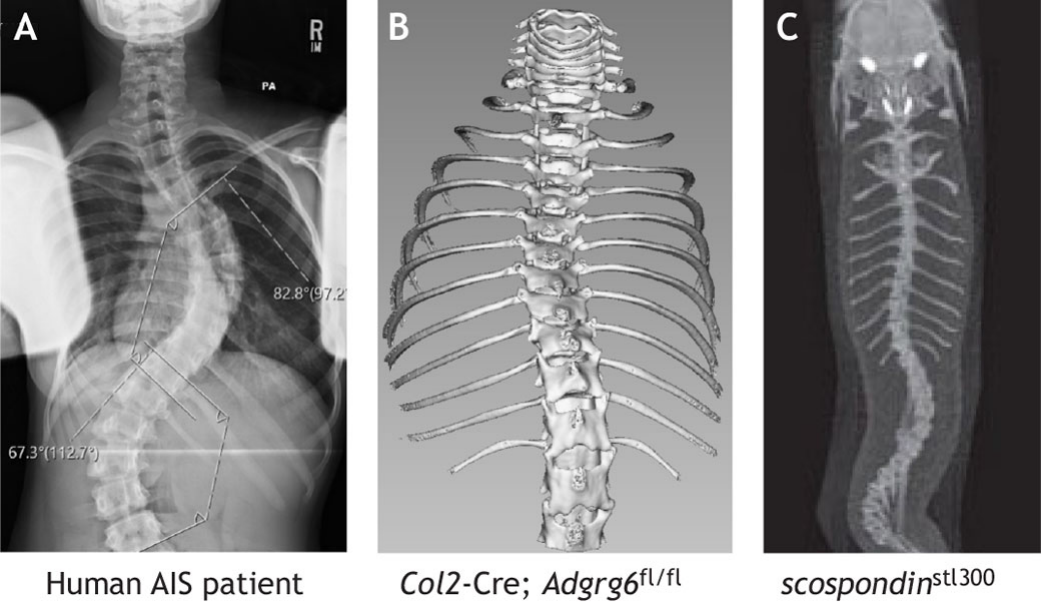
**‘Idiopathic’ spine deformity manifested in human, mouse and zebrafish.** (A) X-ray taken from the back [postero-anterior (PA)] of a patient with typical right AIS, displaying a right thoracic pattern. (B) Micro-computed tomography image of a *Col2a1-*Cre*; Adgrg6^fl/fl^* mouse with scoliosis. (C) X-ray image of a *scospondin^stl300^* mutant zebrafish. Reproduced from Troutwine et al. (2020) with permission from Elsevier. This image is not published under the terms of the CC-BY license of this article. For permission to reuse, please see Troutwine et al. (2020).

Murine and zebrafish models are both valuable experimental tools for investigating human spinal diseases, despite inherent differences most notably in their comparative anatomy. In both mice and zebrafish, the cellular content in the structure connecting the vertebrae, i.e. the intervertebral disc (IVD), differs from that in humans. The number of vertebrae also differs between humans and both mice and zebrafish (Fig. 2A) (Boswell and Ciruna, 2017; Kague et al., 2021; Melrose et al., 2021). In mice, compressive forces along the spine are not proportional to those in humans owing to them being quadrupedal (Melrose et al., 2021). However, the compressive forces in zebrafish spine due to swimming are thought to mimic those along the spinal axis in humans (Boswell and Ciruna, 2017). Although it is important to bear these differences in mind, the ease of genetic modeling in both mice and zebrafish, and the molecular insights each can contribute, justify their use for investigating human scoliosis.

### Mouse models of AIS

The first noted model of idiopathic-like scoliosis was a tissue-specific knockout of adhesion G protein-coupled receptor G6 (*Adgrg6*; previously known as *Gpr126*), orthologous to a gene linked to human AIS (Karner et al., 2015; Kou et al., 2013). Using a constitutive collagen, type II, alpha 1 (*Col2a1*)*-*Cre driver (Long et al., 2001) to remove *Adgrg6* from osteoprogenitor cells produced moderate thoracic scoliosis (Fig. 2B) and/or rib deformation known as pectus excavatum, beginning at postnatal day (P)20 and becoming more prevalent by P120 (Liu et al., 2021). However, the frequency of scoliosis was considerably lower when recombination was induced postnatally, suggesting that ‘idiopathic’ scoliosis has its beginnings in early development. Further studies in which *Adgrg6* was removed from various tissues of the spine (using specific Cre drivers) showed that dense connective tissues including ligaments and parts of the IVD, but not bone, mediate scoliosis in this model. None of these models exhibited sex bias (Liu et al., 2021). Thus, *Adgrg6* genetically engineered mouse models (GEMMs) proved that quadrupeds can model coronal spine deformity, albeit without the sex bias observed in humans (Fig. 1). The *Adgrg6* GEMMs also supported the concept that AIS susceptibility can arise from pre-existing deficiencies in a specific osteoprogenitor cell type early in development.

In human AIS genetic studies, the most robust and replicated GWAS signal occurs in a region flanking the *LBX1* gene (Jiang et al., 2013; Londono et al., 2014; Takahashi et al., 2011). *Lbx1*, encoding the Ladybird homeobox transcription factor first described in *Drosophila*, in mouse determines early myocyte precursor migration, interneuron cell fate and the specification of cardiac neural crest cells (De Graeve et al., 2004; Jagla et al., 1997a,b). Deleting *Lbx1* from muscle precursors using an actin promoter-driven *Cre* transgenic (*Lbx1*^Δmus^) produced mice with progressive kyphosis (a curve in the sagittal plane as opposed to the coronal plane) and hypoplastic forelimbs (Matsuhashi et al., 2023). Other gene-targeting studies in mouse have implicated proprioceptive mechanoreceptors of the muscle in AIS. In one study, removing runt related transcription factor 3 (*Runx3*), which disrupts neurons connecting spinal cord to proprioceptive mechanoreceptors, produced progressive scoliosis during the time of pubertal growth. Further work specifically implicated two types of proprioceptive mechanosensors, the muscle spindle and the Golgi tendon organ (Blecher et al., 2017). Loss of piezo-type mechanosensitive ion channel component 2 (*Piezo2*), another well-described mechanoreceptor, in proprioceptive neurons produces a scoliosis-like deformity in mouse (Assaraf et al., 2020).

A third locus reproducibly associated with AIS is within a non-coding enhancer, *PEC7*, distal to the paired box 1 (*PAX1*) gene (Sharma et al., 2015). Distinct from *Lbx1* and *Adgrg6* loci, the *PEC7* association was specific to female AIS. Genetic knockout of *Pax1* in mice produces the undulated phenotype marked by lumbar vertebral malformations and kinked tail (Adham et al., 2005; Wallin et al., 1994; Balling et al., 1988; Wright, 1947). Deleting orthologous *PEC7* and nearby enhancer *Xe1* in mice conferred a female-biased kinky tail phenotype, whereas deleting *Xe1* alone produced kinky tails equally in males and females (Kokubu et al., 2009; Ushiki et al., 2024). Moreover, the female bias was corrected when pregnant mothers were treated with tamoxifen, implicating estrogen in the kinked tail mechanism (Ushiki et al., 2024). Thus, the *PEC7/Xe1* mouse is the first and only model to pharmacologically demonstrate a role for hormonal receptors in AIS sexual dimorphism.

### Zebrafish models of AIS

Zebrafish (*Danio rerio*) have proven valuable for elucidating the roles of AIS candidate genes identified in human cohorts. In one study, exome-wide analysis of patient genomes identified association with a variant (p. Ala391Thr) in the solute carrier family 39 member 8 (*SLC39A8*) cation transporter gene (Haller et al., 2018). Zebrafish with a *slc39a8* truncating mutation developed vertebral malformations and significantly decreased motor activity. Remarkably, exposure to manganese prevented the phenotype, suggesting a potential dietary therapy for patients with *SCL39A8*-mediated AIS. In another study, a variant in the glycine transporter gene *GLYT1*, encoding SLC6A9, was linked to AIS in an extended family (Wang et al., 2024). In zebrafish, this variant and other orthologous AIS-associated *GLYT1* variants failed to completely rescue deficient glycine uptake in homozygous *slc6a9^m/m^* loss-of-function mutants. Further, *slc6a9^m/m^* mutants showed aberrant left-right signaling in the spinal cord. Spinal curvature in these animals was partially rescued by a glycine receptor antagonist (strychnine) or glycine neutralizer (sodium benzoate) (Wang et al., 2024). These results suggest that cellular homeostasis mediated by solute transporters is key for maintaining a straight spine.

Several zebrafish models that develop idiopathic-like spinal deformities have the potential to inform AIS pathogenesis. These include zebrafish with mutations in *kinesin 6* (*kif6*) (Buchan et al., 2014), *signal transducer and activator of transcription 3* (*stat3*) (Liu et al., 2017), *scospondin* (*sspo*) (Fig. 2C) (Cantaut-Belarif et al., 2020; Rose et al., 2020; Troutwine et al., 2020) and *protein tyrosine kinase 7a* (*ptk7a*) genes (Hayes et al., 2014). The *ptk7a* mutant also exhibits strong bias toward severe curves in female fish, reminiscent of human AIS. Furthermore, genetic removal of normal maternal copies of *ptk7a* in early development produces mutant offspring with vertebral anomalies, suggesting mechanistic overlap between congenital and idiopathic phenotypes (Hayes et al., 2014). Studies of *stat3* (Liu et al., 2017), *sspo* (Troutwine et al., 2020) and *ptk7a* (Rose et al., 2020) zebrafish mutants have implicated neuroinflammation in scoliosis, presumably by triggering disruption of cellular homeostasis during spinal growth. In the case of *ptk7a* mutant zebrafish, scoliosis was reduced by anti-inflammatory drug treatment (Rose et al., 2020; Van Gennip et al., 2018). Another interesting series of experiments showed that zebrafish *urotensin 1* and *urotensin 2* genes, encoding Urp1 and Urp2 proteins, are essential for maintaining a straight spine (Bearce et al., 2022; Gaillard et al., 2023). Urp1 and Urp2 are synthesized by fluid-contacting neurons located in the ventral spinal cord, where they bind to Urotensin-2 receptor 3 (Uts2r3) in dorsal muscles and thereby regulate muscle tone in zebrafish (Egginger et al., 2006; Zhang et al., 2018). With the exception of *ptk7a*, none of these genes themselves have been identified as mutated in AIS patients, but collectively they point to spinal fluid flow and neuroinflammation as important processes in maintaining a straight spine.

An inference from zebrafish screens is that many genetic mutations and biochemical pathways contribute to spine morphogenesis and homeostasis

Zebrafish are also amenable to large forward screens in which N-ethyl N-nitrosourea (ENU) is used to induce mutations in the male germline that may show dominant or recessive inheritance after breeding (Busse et al., 2020). This strategy, combined with complementation testing and genome sequencing, has identified several mutations causing scoliosis. One screen identified scoliosis-causing mutations in *kif6* and *sspo*, which were previously studied as models of spine deformity, thus providing proof of concept for the approach. This productive screen identified in total 40 adult-viable (three dominant and 37 recessive) spine deformity mutations (Gray et al., 2021). An inference from zebrafish screens is that many genetic mutations and biochemical pathways contribute to spine morphogenesis and homeostasis. Missense mutations created by ENU mutagenesis in zebrafish have also uncovered the hypomorphic nature of recessive scoliosis alleles occurring in genes for which complete loss was otherwise lethal (Wise et al., 2020).

## Future directions and challenges

What is the road forward for building pre-clinical models of AIS? Ideally, such models would survive to adulthood and manifest a progressive, growth-correlated spine deformity. Developing multiple, complementary pre-clinical models will be key in the quest to fully represent the molecular inputs controlling spine straightness. In this regard, we note that postnatal phenotyping pipelines are proving fruitful for identifying mouse mutants with skeletal phenotypes including spine deformities (Austin et al., 2004; Rios et al., 2021a,b). A compilation of genes that have been associated with an idiopathic scoliosis-like phenotype, whether in zebrafish, humans or mice, is provided in Table S1. Although we have focused here on mouse and zebrafish, as they are well developed and high throughput for gene discovery and phenotypic assessments, it will also be important to develop other systems such as *ex vivo* spine models or human organoids that could be adapted for rapid drug screening or biomechanical testing. We also note that the advent of artificial intelligence-driven algorithms is expected to accelerate modeling of the genetic and environmental inputs into AIS (Kuznets-Speck et al., 2025).

Either way, we urge researchers to press ahead with creating pre-clinical models that will support molecular understanding and therapeutic testing for AIS and possibly other common disturbances of spine homeostasis

Ultimately, AIS should be best modeled by well-studied vertebrate systems that offer an arsenal of tools for physiological, genetic, environmental, and gene-by-gene or gene-by-environment interaction studies. Both mice and zebrafish arguably offer these benefits, with certain limitations. The evolutionary distance between human and zebrafish or mouse genomes may preclude studying certain alleles. Second, the majority of variants associated with AIS thus far are hypomorphic alleles affecting protein-coding genes (missense or nonsense mutations in C-terminal protein regions) or non-coding regulatory elements (Wise et al., 2020). Although the molecular consequences of these ‘weak’ alleles with small effects are difficult to measure, they may be uncovered by breeding to homozygosity (Gray et al., 2021; Rios et al., 2021b) or, potentially, by combining multiple susceptibility alleles into a single model. Consequently, researchers should accept that the inheritance model that yields a scoliosis phenotype may differ between humans and animal models. Moreover, this strategy could offer the opportunity to measure the molecular effects of hypomorphic variants even in the absence of a full-blown spine deformity phenotype. Thus, some models may inform the AIS ‘molecular phenotype’, while others will be amenable to physiological phenotyping and therapeutic testing (Table S1). Either way, we urge researchers to press ahead with creating pre-clinical models that will support molecular understanding and therapeutic testing for AIS and possibly other common disturbances of spine homeostasis.

Another conclusion to be drawn from ongoing translational studies of AIS is that animal models that do not perfectly re-capitulate human idiopathic-type spinal curvature may be informative nevertheless for deriving biological understanding of the disease. An example is the *Pax1* enhancer deletion strains, in which deleting the orthologous genetic locus associated with human AIS (*PEC7*) in combination with another enhancer leads to a kinky tail without spinal curvature. In this model, a kinked tail served as a proxy for scoliosis (Ushiki et al., 2024). In another example, the observation that the same *ptk7* mutation can produce a congenital or idiopathic phenotype depending on maternal background raises the possibility that the two distinct diagnoses of congenital scoliosis and AIS are genetically related. Such a concept was previously put forth in a study that found congenital scoliosis to be more frequent in families with cases of AIS than in the general population (Grimbacher et al., 1999). Consequently, we expect that additional congenital scoliosis models should inform understanding of idiopathic forms of scoliosis (Giampietro et al., 1999).

It is likely that genetically engineered pre-clinical models will be pushed further toward an AIS phenotype by interactions with hormonal or mechanical stressors (Fig. 1). Regarding the latter, it has been proposed that the cranial to caudal spinal loading that fish experience while swimming mimics the directional forces on the human spine due to walking upright (Boswell and Ciruna, 2017). Upright posture exerts immense stress on the spine, and these forces are exacerbated during periods of growth (Castelein et al., 2020). However, compressive forces are proposed to be even greater in the mouse spine owing to their quadrupedal posture (Melrose et al., 2021). Further investigation is needed to understand how the mechanical forces endured by the adolescent spine are translated into molecular cues. For example, the effects of spinal loading may be tested by allowing zebrafish to swim in fluids of increasing viscosity (Ravel et al., 2025).

Genome engineering using editing methods such as CRISPR or transcription activator-like effector endonucleases (Shamshirgaran et al., 2022) will continue to facilitate production of orthologous AIS-associated alleles that can be tested on their own or in combination in model systems. Tissue-specific Cre-mediated conditional deletion strains will additionally facilitate definition of the tissue and cell type specificity of AIS pathogenesis. Developing pre-clinical models that closely recapitulate human AIS is a major and worthwhile effort toward therapeutic testing, even as the field utilizes ‘less than perfect’ models for basic molecular discoveries.

## Supplementary Material

10.1242/dmm.052438_sup1 Supplementary information

## References

[DMM052438C1] Adham, I. M., Gille, M., Gamel, A. J., Reis, A., Dressel, R., Steding, G., Brand-Saberi, B. and Engel, W. (2005). The scoliosis (sco) mouse: a new allele of Pax1. *Cytogenet Genome Res.* 111, 16-26. 10.1159/000085665 16093716

[DMM052438C2] Asher, M. A. and Burton, D. C. (2006). Adolescent idiopathic scoliosis: natural history and long term treatment effects. *Scoliosis* 1, 2. 10.1186/1748-7161-1-2 16759428 PMC1475645

[DMM052438C3] Assaraf, E., Blecher, R., Heinemann-Yerushalmi, L., Krief, S., Carmel Vinestock, R., Biton, I. E., Brumfeld, V., Rotkopf, R., Avisar, E., Agar, G. et al. (2020). Piezo2 expressed in proprioceptive neurons is essential for skeletal integrity. *Nat. Commun.* 11, 3168. 10.1038/s41467-020-16971-6 32576830 PMC7311488

[DMM052438C4] Austin, C. P., Bacey, J. F., Bradley, A., Bucan, M., Capecchi, M., Collins, F. S., Dove, W. F., Duyk, G., Dymecki, S. and Eppig, J. T. (2004). The knockout mouse project. *Nat. Genet.* 36, 921-924. 10.1038/ng0904-921 15340423 PMC2716027

[DMM052438C5] Balling, R., Deutsch, U. and Gruss, P. (1988). undulated, a mutation affecting the development of the mouse skeleton, has a point mutation in the paired box of Pax 1. *Cell* 55, 531-535. 10.1016/0092-8674(88)90039-6 3180219

[DMM052438C6] Baschal, E. E., Wethey, C. I., Swindle, K., Baschal, R. M., Gowan, K., Tang, N. L. S., Alvarado, D. M., Haller, G. E., Dobbs, M. B., Taylor, M. R. G. et al. (2014). Exome sequencing identifies a rare HSPG2 variant associated with familial idiopathic scoliosis. *G3 (Bethesda)* 5, 167-174. 10.1534/g3.114.015669 25504735 PMC4321025

[DMM052438C7] Bearce, E. A., Irons, Z. H., O'Hara-Smith, J. R., Kuhns, C. J., Fisher, S. I., Crow, W. E. and Grimes, D. T. (2022). Urotensin II-related peptides, Urp1 and Urp2, control zebrafish spine morphology. *eLife* 11, e83883. 10.7554/eLife.83883 36453722 PMC9836392

[DMM052438C8] Blecher, R., Krief, S., Galili, T., Biton, I. E., Stern, T., Assaraf, E., Levanon, D., Appel, E., Anekstein, Y., Agar, G. et al. (2017). The proprioceptive system masterminds spinal alignment: insight into the mechanism of scoliosis. *Dev. Cell* 42, 388-399.e3. 10.1016/j.devcel.2017.07.022 28829946

[DMM052438C9] Boswell, C. W. and Ciruna, B. (2017). Understanding idiopathic scoliosis: a new zebrafish school of thought. *Trends Genet.* 33, 183-196. 10.1016/j.tig.2017.01.001 28174019

[DMM052438C10] Buchan, J. G., Gray, R. S., Gansner, J. M., Alvarado, D. M., Burgert, L., Gitlin, J. D., Gurnett, C. A. and Goldsmith, M. I. (2014). Kinesin family member 6 (kif6) is necessary for spine development in zebrafish. *Dev. Dyn.* 243, 1646-1657. 10.1002/dvdy.24208 25283277 PMC6207368

[DMM052438C11] Busse, B., Galloway, J. L., Gray, R. S., Harris, M. P. and Kwon, R. Y. (2020). Zebrafish: an emerging model for orthopedic research. *J. Orthop. Res.* 38, 925-936. 10.1002/jor.24539 31773769 PMC7162720

[DMM052438C12] Cantaut-Belarif, Y., Orts Del'immagine, A., Penru, M., Pezeron, G., Wyart, C. and Bardet, P. L. (2020). Adrenergic activation modulates the signal from the Reissner fiber to cerebrospinal fluid-contacting neurons during development. *eLife* 9, e59469. 10.7554/eLife.59469 33048048 PMC7591253

[DMM052438C13] Castelein, R. M., Pasha, S., Cheng, J. C. Y. and Dubousset, J. (2020). Idiopathic scoliosis as a rotatory decompensation of the spine. *J. Bone Miner. Res.* 35, 1850-1857. 10.1002/jbmr.4137 32697856

[DMM052438C14] Chan, V., Fong, G. C. Y., Luk, K. D. K., Yip, B., Lee, M.-K., Wong, M.-S., Lu, D. D. S. and Chan, T.-K. (2002). A genetic locus for adolescent idiopathic scoliosis linked to chromosome 19p13.3. *Am. J. Hum. Genet.* 71, 401-406. 10.1086/341607 12094330 PMC379172

[DMM052438C15] Cheng, T., Einarsdottir, E., Kere, J. and Gerdhem, P. (2022). Idiopathic scoliosis: a systematic review and meta-analysis of heritability. *EFORT Open Rev.* 7, 414-421. 10.1530/EOR-22-0026 35638601 PMC9257730

[DMM052438C16] De Graeve, F., Jagla, T., Daponte, J.-P., Rickert, C., Dastugue, B., Urban, J. and Jagla, K. (2004). The ladybird homeobox genes are essential for the specification of a subpopulation of neural cells. *Dev. Biol.* 270, 122-134. 10.1016/j.ydbio.2004.02.014 15136145

[DMM052438C17] Egginger, J.-G., Camus, A. and Calas, A. (2006). Urotensin-II expression in the mouse spinal cord. *J. Chem. Neuroanat.* 31, 146-154. 10.1016/j.jchemneu.2005.10.004 16361078

[DMM052438C18] Einarsdottir, E., Grauers, A., Wang, J., Jiao, H., Escher, S. A., Danielsson, A., Simony, A., Andersen, M., Christensen, S. B., Åkesson, K. et al. (2017). CELSR2 is a candidate susceptibility gene in idiopathic scoliosis. *PLoS ONE* 12, e0189591. 10.1371/journal.pone.0189591 29240829 PMC5730153

[DMM052438C19] Gaillard, A.-L., Mohamad, T., Quan, F. B., de Cian, A., Mosimann, C., Tostivint, H. and Pézeron, G. (2023). Urp1 and Urp2 act redundantly to maintain spine shape in zebrafish larvae. *Dev. Biol.* 496, 36-51. 10.1016/j.ydbio.2023.01.010 36736605

[DMM052438C20] Giampietro, P. F., Raggio, C. L. and Blank, R. D. (1999). Synteny-defined candidate genes for congenital and idiopathic scoliosis. *Am. J. Med. Genet.* 83, 164-177. 10.1002/(SICI)1096-8628(19990319)83:3<164::AID-AJMG5>3.0.CO;2-D 10096591

[DMM052438C21] Gray, R. S., Gonzalez, R., Ackerman, S. D., Minowa, R., Griest, J. F., Bayrak, M. N., Troutwine, B., Canter, S., Monk, K. R., Sepich, D. S. et al. (2021). Postembryonic screen for mutations affecting spine development in zebrafish. *Dev. Biol.* 471, 18-33. 10.1016/j.ydbio.2020.11.009 33290818 PMC10785604

[DMM052438C22] Grimbacher, B., Holland, S. M., Gallin, J. I., Greenberg, F., Hill, S. C., Malech, H. L., Miller, J. A., O'Connell, A. C. and Puck, J. M. (1999). Hyper-IgE syndrome with recurrent infections--an autosomal dominant multisystem disorder. *N. Engl. J. Med.* 340, 692-702. 10.1056/NEJM199903043400904 10053178

[DMM052438C23] Haller, G., Alvarado, D., McCall, K., Yang, P., Cruchaga, C., Harms, M., Goate, A., Willing, M., Morcuende, J. A., Baschal, E. et al. (2016). A polygenic burden of rare variants across extracellular matrix genes among individuals with adolescent idiopathic scoliosis. *Hum. Mol. Genet.* 25, 202-209. 10.1093/hmg/ddv463 26566670 PMC4690498

[DMM052438C24] Haller, G., McCall, K., Jenkitkasemwong, S., Sadler, B., Antunes, L., Nikolov, M., Whittle, J., Upshaw, Z., Shin, J., Baschal, E. et al. (2018). A missense variant in SLC39A8 is associated with severe idiopathic scoliosis. *Nat. Commun.* 9, 4171. 10.1038/s41467-018-06705-0 30301978 PMC6177404

[DMM052438C25] Hayes, M., Gao, X., Yu, L. X., Paria, N., Henkelman, R. M., Wise, C. A. and Ciruna, B. (2014). ptk7 mutant zebrafish models of congenital and idiopathic scoliosis implicate dysregulated Wnt signalling in disease. *Nat. Commun.* 5, 4777. 10.1038/ncomms5777 25182715 PMC4155517

[DMM052438C27] Hresko, M. T. (2013). Clinical practice. Idiopathic scoliosis in adolescents. *N. Engl. J. Med.* 368, 834-841. 10.1056/NEJMcp1209063 23445094

[DMM052438C28] Jagla, K., Frasch, M., Jagla, T., Dretzen, G., Bellard, F. and Bellard, M. (1997a). ladybird, a new component of the cardiogenic pathway in Drosophila required for diversification of heart precursors. *Development* 124, 3471-3479. 10.1242/dev.124.18.3471 9342040

[DMM052438C29] Jagla, K., Jagla, T., Heitzler, P., Dretzen, G., Bellard, F. and Bellard, M. (1997b). ladybird, a tandem of homeobox genes that maintain late wingless expression in terminal and dorsal epidermis of the Drosophila embryo. *Development* 124, 91-100. 10.1242/dev.124.1.91 9006070

[DMM052438C30] Jiang, H., Qiu, X., Dai, J., Yan, H., Zhu, Z., Qian, B. and Qiu, Y. (2013). Association of rs11190870 near LBX1 with adolescent idiopathic scoliosis susceptibility in a Han Chinese population. *Eur. Spine J.* 22, 282-286. 10.1007/s00586-012-2532-4 23096252 PMC3555620

[DMM052438C31] Kague, E., Turci, F., Newman, E., Yang, Y., Brown, K. R., Aglan, M. S., Otaify, G. A., Temtamy, S. A., Ruiz-Perez, V. L., Cross, S. et al. (2021). 3D assessment of intervertebral disc degeneration in zebrafish identifies changes in bone density that prime disc disease. *Bone Res.* 9, 39. 10.1038/s41413-021-00156-y 34465741 PMC8408153

[DMM052438C32] Karner, C. M., Long, F., Solnica-Krezel, L., Monk, K. R. and Gray, R. S. (2015). Gpr126/Adgrg6 deletion in cartilage models idiopathic scoliosis and pectus excavatum in mice. *Hum. Mol. Genet.* 24, 4365-4373. 10.1093/hmg/ddv170 25954032 PMC4492399

[DMM052438C33] Karol, L. A., Johnston, C. E., II, Browne, R. H. and Madison, M. (1993). Progression of the curve in boys who have idiopathic scoliosis. *J. Bone Joint Surg. Am.* 75, 1804-1810. 10.2106/00004623-199312000-00010 8258551

[DMM052438C34] Khanshour, A. M., Kou, I., Fan, Y., Einarsdottir, E., Makki, N., Kidane, Y. H., Kere, J., Grauers, A., Johnson, T. A., Paria, N. et al. (2018). Genome-wide meta-analysis and replication studies in multiple ethnicities identify novel adolescent idiopathic scoliosis susceptibility loci. *Hum. Mol. Genet.* 27, 3986-3998. 10.1093/hmg/ddy306 30395268 PMC6488972

[DMM052438C35] Kokubu, C., Horie, K., Abe, K., Ikeda, R., Mizuno, S., Uno, Y., Ogiwara, S., Ohtsuka, M., Isotani, A., Okabe, M. et al. (2009). A transposon-based chromosomal engineering method to survey a large cis-regulatory landscape in mice. *Nat. Genet.* 41, 946-952. 10.1038/ng.397 19633672

[DMM052438C36] Kou, I., Takahashi, Y., Johnson, T. A., Takahashi, A., Guo, L., Dai, J., Qiu, X., Sharma, S., Takimoto, A., Ogura, Y. et al. (2013). Genetic variants in GPR126 are associated with adolescent idiopathic scoliosis. *Nat. Genet.* 45, 676-679. 10.1038/ng.2639 23666238

[DMM052438C37] Kou, I., Otomo, N., Takeda, K., Momozawa, Y., Lu, H.-F., Kubo, M., Kamatani, Y., Ogura, Y., Takahashi, Y., Nakajima, M. et al. (2019). Genome-wide association study identifies 14 previously unreported susceptibility loci for adolescent idiopathic scoliosis in Japanese. *Nat. Commun.* 10, 3685. 10.1038/s41467-019-11596-w 31417091 PMC6695451

[DMM052438C38] Kuznets-Speck, B., Ogonor, B. K., Wytock, T. P. and Motter, A. E. (2025). Generative prediction of causal gene sets responsible for complex traits. *Proc. Natl. Acad. Sci. USA* 122, e2415071122. 10.1073/pnas.2415071122 40504147 PMC12184495

[DMM052438C39] Little, D. G., Song, K. M., Katz, D. and Herring, J. A. (2000). Relationship of peak height velocity to other maturity indicators in idiopathic scoliosis in girls. *J. Bone Joint Surg.* 82, 685-693. 10.2106/00004623-200005000-00009 10819279

[DMM052438C40] Liu, Y., Sepich, D. S. and Solnica-Krezel, L. (2017). Stat3/Cdc25a-dependent cell proliferation promotes embryonic axis extension during zebrafish gastrulation. *PLoS Genet.* 13, e1006564. 10.1371/journal.pgen.1006564 28222105 PMC5319674

[DMM052438C41] Liu, Z., Hussien, A. A., Wang, Y., Heckmann, T., Gonzalez, R., Karner, C. M., Snedeker, J. G. and Gray, R. S. (2021). An adhesion G protein-coupled receptor is required in cartilaginous and dense connective tissues to maintain spine alignment. *eLife* 10, e67781. 10.7554/eLife.67781.sa2 34318745 PMC8328515

[DMM052438C42] Londono, D., Kou, I., Johnson, T. A., Sharma, S., Ogura, Y., Tsunoda, T., Takahashi, A., Matsumoto, M., Herring, J. A., Lam, T.-P. et al. (2014). A meta-analysis identifies adolescent idiopathic scoliosis association with LBX1 locus in multiple ethnic groups. *J. Med. Genet.* 51, 401-406. 10.1136/jmedgenet-2013-102067 24721834

[DMM052438C43] Long, F., Zhang, X. M., Karp, S., Yang, Y. and McMahon, A. P. (2001). Genetic manipulation of hedgehog signaling in the endochondral skeleton reveals a direct role in the regulation of chondrocyte proliferation. *Development* 128, 5099-5108. 10.1242/dev.128.24.5099 11748145

[DMM052438C44] Matsuhashi, Y., Horiuchi, K., Nakagawa, T., Takahashi, Y., Imabayashi, H., Hosogane, N., Watanabe, K., Matsumoto, M. and Chiba, K. (2023). Abrogation of LBX1 in skeletal muscle results in hypoplastic limbs and progressive kyphosis in mice. *J. Orthop. Res.* 41, 884-890. 10.1002/jor.25417 35856296

[DMM052438C45] Melrose, J., Tessier, S. and Risbud, M. V. (2021). Genetic murine models of spinal development and degeneration provide valuable insights into intervertebral disc pathobiology. *Eur. Cell Mater.* 41, 52-72. 10.22203/eCM.v041a05 33432564

[DMM052438C46] Murphy, R. F. and Mooney, J. F., III. (2016). Complications following spine fusion for adolescent idiopathic scoliosis. *Curr. Rev. Musculoskelet. Med.* 9, 462-469. 10.1007/s12178-016-9372-5 27639726 PMC5127952

[DMM052438C47] Patten, S. A., Margaritte-Jeannin, P., Bernard, J.-C., Alix, E., Labalme, A., Besson, A., Girard, S. L., Fendri, K., Fraisse, N., Biot, B. et al. (2015). Functional variants of POC5 identified in patients with idiopathic scoliosis. *J. Clin. Invest.* 125, 1124-1128. 10.1172/JCI77262 25642776 PMC4362221

[DMM052438C48] Ravel, G., Mercé, T., Bergmann, M., Knoll-Gellida, A., Bouharguane, A., Al Kassir, S., Iollo, A. and Babin, P. J. (2025). Modeling zebrafish escape swim reveals maximum neuromuscular power output and efficient body movement adaptation to increased water viscosity. *iScience* 28, 112056. 10.1016/j.isci.2025.112056 40124491 PMC11930232

[DMM052438C49] Richards, B. S., Sucato, D. J. and Johnston, C. E. (2020). Scoliosis. In *Tachdjian's Pediatric Orthopaedics*, Vol. 1 (ed. J. A. Herring), pp. 209-307. Elsevier.

[DMM052438C50] Rios, J. J., Denton, K., Russell, J., Kozlitina, J., Ferreira, C. R., Lewanda, A. F., Mayfield, J. E., Moresco, E., Ludwig, S., Tang, M. et al. (2021a). Germline saturation mutagenesis induces skeletal phenotypes in mice. *J. Bone Miner. Res.* 36, 1548-1565. 10.1002/jbmr.4323 33905568 PMC8862308

[DMM052438C51] Rios, J. J., Denton, K., Yu, H., Manickam, K., Garner, S., Russell, J., Ludwig, S., Rosenfeld, J. A., Liu, P., Munch, J. et al. (2021b). Saturation mutagenesis defines novel mouse models of severe spine deformity. *Dis. Model. Mech.* 14, dmm048901. 10.1242/dmm.048901 34142127 PMC8246263

[DMM052438C52] Rose, C. D., Pompili, D., Henke, K., Van Gennip, J. L. M., Meyer-Miner, A., Rana, R., Gobron, S., Harris, M. P., Nitz, M. and Ciruna, B. (2020). SCO-spondin defects and neuroinflammation are conserved mechanisms driving spinal deformity across genetic models of idiopathic scoliosis. *Curr. Biol.* 30, 2363-2373.e6. 10.1016/j.cub.2020.04.020 32386528

[DMM052438C53] Shamshirgaran, Y., Liu, J., Sumer, H., Verma, P. J. and Taheri-Ghahfarokhi, A. (2022). Tools for efficient genome editing; ZFN, TALEN, and CRISPR. *Methods Mol. Biol.* 2495, 29-46. 10.1007/978-1-0716-2301-5_2 35696026

[DMM052438C54] Sharma, S., Londono, D., Eckalbar, W. L., Gao, X., Zhang, D., Mauldin, K., Kou, I., Takahashi, A., Matsumoto, M., Kamiya, N. et al. (2015). A PAX1 enhancer locus is associated with susceptibility to idiopathic scoliosis in females. *Nat. Commun.* 6, 6452. 10.1038/ncomms7452 25784220 PMC4365504

[DMM052438C55] Takahashi, Y., Kou, I., Takahashi, A., Johnson, T. A., Kono, K., Kawakami, N., Uno, K., Ito, M., Minami, S., Yanagida, H. et al. (2011). A genome-wide association study identifies common variants near LBX1 associated with adolescent idiopathic scoliosis. *Nat. Genet.* 43, 1237-1240. 10.1038/ng.974 22019779

[DMM052438C56] Tang, N. L. S., Yeung, H. Y., Hung, V. W. Y., Di Liao, C., Lam, T. P., Yeung, H. M., Lee, K. M., Ng, B. K. W. and Cheng, J. C. Y. (2012). Genetic epidemiology and heritability of AIS: a study of 415 Chinese female patients. *J. Orthop. Res.* 30, 1464-1469. 10.1002/jor.22090 22362628

[DMM052438C57] Tang, N. L. S., Dobbs, M. B., Gurnett, C. A., Qiu, Y., Lam, T. P., Cheng, J. C. Y. and Hadley- Miller, N. (2021). A decade in review after idiopathic scoliosis was first called a complex trait- a tribute to the Late Dr. Yves Cotrel for his support in studies of etiology of scoliosis. *Genes (Basel)* 12, 1033. 10.3390/genes12071033 34356049 PMC8306836

[DMM052438C58] Troutwine, B. R., Gontarz, P., Konjikusic, M. J., Minowa, R., Monstad-Rios, A., Sepich, D. S., Kwon, R. Y., Solnica-Krezel, L. and Gray, R. S. (2020). The Reissner fiber is highly dynamic *in vivo* and controls morphogenesis of the spine. *Curr. Biol.* 30, 2353-2362.e3. 10.1016/j.cub.2020.04.015 32386529 PMC7891109

[DMM052438C59] Ushiki, A., Sheng, R. R., Zhang, Y., Zhao, J., Nobuhara, M., Murray, E., Ruan, X., Rios, J. J., Wise, C. A. and Ahituv, N. (2024). Deletion of Pax1 scoliosis-associated regulatory elements leads to a female-biased tail abnormality. *Cell Rep.* 43, 113907. 10.1016/j.celrep.2024.113907 38461417 PMC11005513

[DMM052438C60] Van Gennip, J. L. M., Boswell, C. W. and Ciruna, B. (2018). Neuroinflammatory signals drive spinal curve formation in zebrafish models of idiopathic scoliosis. *Sci. Adv.* 4, eaav1781. 10.1126/sciadv.aav1781 30547092 PMC6291318

[DMM052438C61] Wallin, J., Wilting, J., Koseki, H., Fritsch, R., Christ, B. and Balling, R. (1994). The role of Pax-1 in axial skeleton development. *Development* 120, 1109-1121. 10.1242/dev.120.5.1109 8026324

[DMM052438C62] Wang, X., Yue, M., Cheung, J. P. Y., Cheung, P. W. H., Fan, Y., Wu, M., Wang, X., Zhao, S., Khanshour, A. M., Rios, J. J. et al. (2024). Impaired glycine neurotransmission causes adolescent idiopathic scoliosis. *J. Clin. Invest.* 134, e168783. 10.1172/JCI168783 37962965 PMC10786698

[DMM052438C63] Weinstein, S. L., Dolan, L. A., Cheng, J. C. Y., Danielsson, A. and Morcuende, J. A. (2008). Adolescent idiopathic scoliosis. *The Lancet* 371, 1527-1537. 10.1016/S0140-6736(08)60658-3 18456103

[DMM052438C64] Wise, C. A., Sepich, D., Ushiki, A., Khanshour, A. M., Kidane, Y. H., Makki, N., Gurnett, C. A., Gray, R. S., Rios, J. J., Ahituv, N. et al. (2020). The cartilage matrisome in adolescent idiopathic scoliosis. *Bone Res.* 8, 13. 10.1038/s41413-020-0089-0 32195011 PMC7062733

[DMM052438C65] Wright, M. E. (1947). Undulated: a new genetic factor in Mus musculus affecting the spine and tail. *Heredity* 1, 137-141. 10.1038/hdy.1947.10

[DMM052438C66] Yu, H., Khanshour, A. M., Ushiki, A., Otomo, N., Koike, Y., Einarsdottir, E., Fan, Y., Antunes, L., Kidane, Y. H., Cornelia, R. et al. (2024). Association of genetic variation in COL11A1 with adolescent idiopathic scoliosis. *eLife* 12, RP89762. 10.7554/eLife.89762 38277211 PMC10945706

[DMM052438C67] Zhang, X., Jia, S., Chen, Z., Chong, Y. L., Xie, H., Feng, D., Wu, X., Song, D. Z., Roy, S. and Zhao, C. (2018). Cilia-driven cerebrospinal fluid flow directs expression of urotensin neuropeptides to straighten the vertebrate body axis. *Nat. Genet.* 50, 1666-1673. 10.1038/s41588-018-0260-3 30420648

