# Supplementary material for: The importance of imperfect pre-clinical models in adolescent idiopathic scoliosis
Source: Dis Model Mech. 2025 Sep 1;18(8):dmm052438. doi: 10.1242/dmm.052438 (PMC12452065; doi:10.1242/dmm.052438)
Supplement: Supplementary information [file dmm-18-052438-s1.pdf]

Table S1. Genes identified with an idiopathic scoliosis phenotype in humans, zebrafish or mice

**Gene**	**Human gene**<br><br>**Disease (OMIM #)**	**Zebrafish gene**<br><br>**Phenotype (Zfin #)**	**Mouse gene**<br><br>**Phenotype (MGI #)**	**Citation**<br><br>**(Z-zebrafish, H-human, M-mouse)**
ATP-binding cassette, sub-family C (CFTR/MRP), member 6a)	***ABCC6***<br><br>pseudoxanthoma elasticum<br><br>(recessive) (264800)	***abcc6a***<br><br>scoliosis, excess bone deposition by 6 mpf<br><br>no data for ***abcc6b*** ortholog	***Abcc6***<br><br>Null mice show patchy mineralization in areas including arteries, skin, retina, kidney, and interscapular brown fat<br><br>(1351634)	Z (Van Gils, Willaert, De Vilder, Coucke, & Vanakker, 2018)
ABO, alpha 1-3-N-acetylgalactosaminyltransferase and alpha 1-3-galactosyltranferase	***ABO***<br><br>GWAS AIS<br><br>(110300)	***No direct ortholog***	***Abo***<br><br>(2135738)	H (Khanshour et al., 2018)
ADAM metallopeptidase with thrombospondin type 1 motif, 9)	***ADAMTS9***<br><br>Nephronophthisis<br><br>(605421)	***adamts9***<br><br>caudal or multibend scoliosis, swollen centra canal	***Adamts9***<br><br>KO shows severe developmental defects; early embryonic lethality (1916320)	Z (Gray et al., 2021)
Adhesion G Protein-Coupled Receptor G6	***ADGRG6***<br><br>GWAS AIS	***adgrg6***<br><br>no scoliosis	***Adgrg6***<br><br>KO is embryonic lethal during	Z, H (Kou et al., 2013)<br><br>M (Karner, Long, Solnica-Krezel,

Disease Models & Mechanisms • Supplementary information

	Lethal congenital contracture syndrome 9 (recessive)(616503)	phenotype, delayed ossification of centra	organogenesis (1916151)<br><br>cKO shows scoliosis; pectus excavatum	Monk, & Gray, 2015)
Arachidonase-5 lipoxygenase	***ALOX5***	***alox5a***<br><br>no data	***Alox5***<br><br>Null mice show altered inflammatory responses (87999)<br><br>Homozygous missense allele shows spine deformity	M (Rios et al., 2021)
Armadillo repeat containing 9	***ARMC9***<br><br>Joubert syndrome (recessive)<br><br>(617622)	***armc9***<br><br>multiple bend scoliosis	***Armc9***<br><br>(1926045)	Z (Van De Weghe et al., 2017)
Branched chain keto acid dehydrogenase E1 subunit beta	***BCKDHB/FAM46A***<br><br>GWAS AIS<br><br>Maple syrup urine disease, type Ib (recessive) (620698)	***bckdhb***<br><br>no data	***Bckdhb***<br><br>KO shows early lethality; surviving mice show severe growth delay and biochemical Maple syrup urine disease (88137)	H (Kou et al., 2019)<br><br>M (Pontoizeau et al., 2024)
Basonuclin 2	***BNC2***<br><br>GWAS AIS<br><br>Lower urinary tract obstruction	***bnc2***<br><br>Pigmentation, oogenesis, urinary tract defects; OE gives an early	***Bnc2***<br><br>KO shows severe dwarfism, craniofacial defects (2443805)	H Z (Ogura et al., 2015)

Disease Models & Mechanisms • Supplementary information

	(dominant) (618612)	truncated tail		
Bardet-Biedl syndrome 2	***BBS2***<br><br>BBS2 syndrome and retinal dystrophy (recessive) (615981)	***bbs2***<br><br>Cilia defects and scoliosis	***Bbs2***<br><br>KO mice model BBS2 syndrome (2135267)	Z (Lindstrand et al., 2016)<br><br>Z (Song, Fogerty, Cianciolo, Stupay, & Perkins, 2020)
Coiled-coil domain containing 57	***CCDC57***	***ccdc57***<br><br>Multiple bend scoliosis, forward screen	***Ccdc57***<br><br>Cranial and skeletal anomalies (1918526)	Z (Li et al., 2023)
Cadherin 13	***CDH13***<br><br>GWAS AIS	***cdh13***<br><br>no apparent scoliosis phenotype in LOF allele sa23253	***Cdh13***<br><br>KO mice are morphologically normal and show increased synaptic inhibition (99551)	H (Khanshour et al., 2018)<br><br>M (Rivero et al., 2013~~5~~)
Cilia and flagella associated protein 298	***CFAP298***<br><br>Primary Ciliary Dyskinesia (recessive) (615500)	***cfap298***<br><br>scoliosis	***Cfap298***<br><br>Cilia defects; multiple developmental anomalies (1915251)	Z (Jaffe et al., 2016)
Chloride voltage-gated channel 1	***CLCN1***<br><br>Myotonia congenita (dominant,	***clcn1a/b***	***Clcn1***<br><br>Mutant mice exhibit mild to severe spasms of the hind limbs and abnormal hind limb reflexes.	M (Rios et al., 2021)

	recessive) (160800, 255700)		(88417)<br><br>Homozygous missense allele shows spine deformity	
Collagen type I alpha 1a	***COL1A1***<br><br>Ehlers-Danlos syndrome, osteogenesis imperfecta, subperiosteal bone growth (dominant)<br><br>(114000;619115;130060;166200;166210;259420;166220)	***col1a1a***<br><br>variable vertebral malformations	***Col1a1***<br><br>Various mutants phenotype osteogenesis and other organ defects (88467)	Z (Gistelinck et al., 2018)
Collagen type I alpha 1b	***COL1A1***<br><br>Ehlers-Danlos syndrome, osteogenesis imperfecta, subperiosteal bone growth	***col1a1b***<br><br>variable vertebral malformations	***Col1a1***<br><br>Various mutants phenotype osteogenesis and other organ defects (88467)	Z (Gistelinck et al., 2018)
Collagen type I alpha 2	***COL1A2***<br><br>Osteogenesis imperfecta w/scoliosis (dominant) (166210, 259420,166220);	***col1a2***<br><br>short stature, reduced vertebral arches	***Col1a2***<br><br>Homozygous nulls show multiple bone and connective tissue abnormalities including scoliosis (88468)	Z (Gistelinck et al., 2018; Henke et al., 2017)

Disease Models & Mechanisms • Supplementary information

	Ehlers-Danlos syndromes (619120, 617821, 225320)			
Collagen type II alpha 1a	***COL2A1***<br><br>Stickler syndrome (dominant) (108300; 609508)	***col2a1a***<br><br>vertebral fusions	***Col2a1***<br><br>Homozygous knockout mice can have skeletal fractures, limb deformities, generalized osteopenia, and small body size (88452)	Z (Henke et al., 2017)
Collagen type VIII alpha 1a	***COL8A1***	***col8a1a***<br><br>congenital vertebral malformations secondary to notochord defects	***Col8a1***<br><br>Knockout mice show eye abnormalities (88463)	Z (Gray et al., 2014)
Collagen type X alpha 1 chain	***COL10A1***<br><br>Metaphyseal chondrodysplasia, Schmidt type (dominant) (156500)	***col10a1a***<br><br>kinked tail, reduced TMD, shorter centra length and thickness	***Col10a1***<br><br>Impaired endochondral ossification and hematopoiesis (88445)	Z (Raman et al., 2024)
Collagen type XI alpha 1 chain	***COL11A1***<br><br>GWAS AIS<br><br>Stickler syndrome II, Marshall syndrome	***col11a1a***<br><br>abnormal Meckel's cartilage, otoliths, body length	***Col11a1***<br><br>Knockout mice are chondrodysplastic with flattened vertebrae (88446)	H (Yu et al., 2024)<br><br>M (Li et al., 1995)

Disease Models & Mechanisms • Supplementary information

	(dominant) (604841,154780)<br><br>Fibrochondrogenesis 1(recessive) (228520)<br><br>Deafness, 37 (dominant) (618533)			
Collagen type XI alpha 2 chain	***COL11A2***<br><br>Associated with AIS<br><br>Fibrochondrogenesis 2 (dominant, recessive) (614524); Otospondylomega epiphyseal dysplasia, (dominant) (184840), (recessive) (215150)	***col11a2***<br><br>Vertebral fusions	***Col11a2***<br><br>KO mice show milder phenotype than Col11a1 KO, with smaller body size, receding snouts, and deafness (88447)	Z, H (Haller et al., 2016; Rebello et al., 2023)
cAMP responsive element binding protein 5	***CREB5***<br><br>GWAS AIS	***creb5a/b***<br><br>no data	***Creb5***<br><br>KO is neonatal lethal (2443973)	H (Kou et al., 2019)
CUB and SUSHI multiple domains 1	***CSMD1***<br><br>GWAS AIS	***csmd1a/b***	***Csmd1***<br><br>Abnormal thoracic cage; neuropsychological deficits	H (Kou et al., 2019)

Disease Models & Mechanisms • Supplementary information

			(2137383)	
DeltaB	***DLL1***<br><br>Neurodevelopmental disorder (618709)	***dlb***<br><br>lateral line defects	***Dll1***<br><br>Homozygous null embryos do not survive, lack cranio-caudal polarity and segmented spine (104659)	Z (Lleras-Forero et al., 2020)<br><br>M (Shapiro, 2016)
DeltaC	***DLL3***<br><br>Spondylocostal dysostosis 1 (recessive) (277300)	***dlc***<br><br>fused somites, see tbx6 also	***Dll3***<br><br>Rib, spine, IVD deformities (1096877)	Z (Lleras-Forero et al., 2020)<br><br>M (Shapiro, 2016)
Distal-less homeobox 3b	***DLX3***<br><br>Amelogenesis imperfecta type IV (dominant)(104510)<br><br>Trichodontoosseous syndrome (dominant)(190320)	***dlx3b***<br><br>fused somites, fused vertebrae	***Dlx3***<br><br>Mouse KO is perinatal lethal (94903)	Z (Pang et al., 2020)<br><br>H (Zhao et al., 2016)
Dual serine/threonine and tyrosine protein kinase	***DSTYK***<br><br>renal defects (dominant) (610805), spastic paraplegia (recessive)(270250	***dstyk***<br><br>notochord templated vertebral malformations	***Dstyk***<br><br>Mouse KO is morphologically normal with impaired learning (1925064)	Z (Bagwell et al., 2020)

	)			
Ectonucleotide pyrophosphatase/phosphodiesterase 1	***ENPP1***<br><br>Hypophosphatemic rickets (recessive) (6133120)<br><br>Arterial calcification of infancy (recessive) (208000)	***enpp1***<br><br>pathological mineralization, vertebral fusions	***Enpp1***<br><br>Mouse KO shows abnormal calcifications and scoliosis (97370)	Z (Apschner, Huitema, Ponsioen, Peterson-Maduro, & Schulte-Merker, 2014; Nitschke et al., 2012)
FAT atypical cadherin 4	***FAT4***<br><br>Van Maldergem syndrome 2<br><br>(recessive) (615546)	***fat4***<br><br>no data	***Fat4***<br><br>Homozygous null shows neonatal lethality, reduced birth body size, curly tails, kyphosis, defects in sternum and vertebrae morphology, other organ defects (3045256)<br><br>Homozygous missense allele shows spine deformity	M (Rios et al., 2021)
Fibroblast growth factor 20	***FGF20***	***fgf20 a or b***<br><br>growth of cranium and jaw	***Fgf20***<br><br>Homozygous null shows ear defects (1891346)<br><br>Homozygous missense allele shows spine deformity	Z (Cooper, Wirgau, Sweet, & Albertson, 2013; Yamauchi, Goto, Katayama, Miyake, & Itoh, 2011)<br><br>M (Rios et al., 2021)
FTO alpha-ketogluturate dependent	***FTO***<br><br>GWAS AIS	***fto***<br><br>cilia in pronephros disorganized, jaw	***Fto***<br><br>Mouse KO shows postnatal growth retardation, altered metabolism,	Z (Osborn et al., 2014)

Disease Models & Mechanisms • Supplementary information

dioxygenase	Growth retardation, developmental delay, facial dysmorphism (recessive) (612938)	reduced	and decreased body length (1347093)	
Galactosylceramidase	***GALC***<br><br>Krabbe disease (recessive) (245200)	***galc a,b***<br><br>no data	***Galc***<br><br>Homozygous nulls show tremors, progressive weakness, wasting, demyelination, early death (95636)<br><br>Homozygous missense allele shows spine deformity	M (Rios et al., 2021)
Hairy-related 1	***HES7***<br><br>Spondylocostal dysostosis 4 (recessive) (613686)	***her1***<br><br>Somitogenesis defects	***Hes7***<br><br>Homozygotes nulls show somitogenesis, skeletal defects, trunk and tail, reduced numbers of ribs, vertebral malformation, early death (2135679)<br><br>Homozygous missense allele shows spine deformity	Z (Lleras-Forero et al., 2020)
Hairy and enhancer of split related-7	***HES7***<br><br>Spondylocostal dysostosis 4 recessive (613686)	***her7***<br><br>Somitogenesis	***Hes7***	Z (Lleras-Forero et al., 2020)<br><br>M (Rios et al., 2021)

Disease Models & Mechanisms • Supplementary information

Heterogeneous nuclear ribo nucleoprotein U-like 1	***HNRNPUL1***<br><br>Candidate gene for congenital limb malformations	***hnrnpul1***<br><br>Fin muscle+bone defects, spine caudal side to side bends	***Hnrnpul1***<br><br>(2443517)	Z, H (Blackwell et al., 2022)~~2)~~
Isocitrate dehydrogenase (NADP(+)) 2	***IDH2***<br><br>D-2-hydroxyglutaric aciduria 2 (dominant) (613657)	***idh2***<br><br>no data	***Idh2***<br><br>spinal deformity with age<br><br>(96414)	M (Chae et al., 2018)
Inositol polyphosphate phosphatase-like 1a	***INPPL1***<br><br>Opsismodysplasia (recessive) (248480)	***inppl1a***<br><br>rostral scoliosis	***Inppl1***<br><br>Homozygous nulls show decreased postnatal growth and resistance to diet-induced obesity (1333787)	Z (Fradet & Fitzgerald, 2017)~~)~~
Inositol polyphosphate-5- polyphosphatase D	***INPP5D***	***inpp5d***<br><br>no data	***Inpp5d***<br><br>Homozygous mice show wasting and immunologic defects (107357)<br><br>Homozygous missense allele shows spine deformity	M (Rios et al., 2021)
Importin 8	***IPO8***<br><br>VISS syndrome (recessive) (619472)	***ipo8***<br><br>Dorsoventral and cardiovascular defects	***Ipo8***<br><br>Homozygous null shows Neurologic, eye, hematopoietic defects (2444611)<br><br>Homozygous missense allele shows	M (Rios et al., 2021)

			spine deformity	
Interferon regulatory factor	***IFR8***<br><br>Immunodeficiency 32A, mycobacteriosis (dominant)(614893);<br>Immunodeficiency 32B, mycobacteriosis, (recessive) (226990)	***irf8***<br><br>macrophage development	***Irf8***<br><br>Deregulated hematopoiesis (96395)<br><br>Homozygous missense allele shows spine deformity	M (Rios et al., 2021)
Kinesin family member 7	***KIF7***<br><br>AIS<br><br>Acrocallosal syndrome (recessive) (200900) Joubert syndrome 12 (recessive) (200900)	***kif7***<br><br>Scoliosis, neuro inflammation	***Kif7***<br><br>Homozygous nulls show neonatal lethality with multiple developmental anomalies (1098239)	Z H (Terhune et al., 2021)
Kinesin family member 24	***KIF24***<br><br>GWAS AIS	***n/a***	***Kif24***<br><br>(1918345)	H (Kuo et al., 2019)
Klotho	***KL***	***kl***<br><br>bulbus arteriosa calcification	***Kl***<br><br>Homozygous null mice show short lifespan and growth retardation and abnormal bone (1101771)	M (Rios et al., 2021)

Disease Models & Mechanisms • Supplementary information

			Homozygous missense allele shows spine deformity	
Xylosyl- and glucuronyltransferase 1	***LARGE1***<br><br>Muscular dystrophy-dystroglycanopathy types A,6 (613154);and B,6 (608840) (both recessive)	***large1***<br><br>no data	***Large 1***<br><br>Homozygous nulls show progressive thoracic kyphosis and myopathy, calcium deposits in muscle, loss of Schwann cells and myelin, eye and CNS defects, deafness, reduced growth, early death (342270)<br><br>Homozygous missense allele shows spine deformity	M (Rios et al., 2021)
Ladybird homeobox 1a	***LBX1***<br><br>GWAS AIS	***lbx1a/b***<br>Interneuron fate specification	***Lbx1***<br><br>Homozygous null shows limb muscle, heart, and neurologic defects (104867)<br><br>Deletion of AIS-associated enhancer causes vertebral rotations	Z (Juarez-Morales et al., 2021)<br><br>M (McCallum-Loudeac et al., 2024)<br><br>H (Takahashi et al., 2011)
Latent transforming growth factor beta binding protein 1	***LTBP1***<br><br>Cutis laxa, type IIE (recessive) (619451)	***ltbp1***<br><br>overgrowth on vertebral arches, disordered collagen	***Ltbp1***<br><br>Homozygous nulls show aortic and outflow tract defects and early death (109151)	Z H (Pottie et al., 2021)
Mitogen-activated protein kinase7	***MAPK7***<br><br>AIS	***mapk7***<br><br>scoliosis	***Mapk7***<br><br>Homozygous null shows multiple	Z H (Gao et al., 2017)

Disease Models & Mechanisms • Supplementary information

	Cardiofaciocutaneous syndrome 3 (dominant) (615279)		developmental anomalies and embryonic death ***(***1346347)	
Mesenchyme homeobox 1	***MEOX1***<br><br>Klippel-Feil syndrome 2 (recessive) (214300)	***meox1***<br><br>fusion of vertebra at mineralization, overgrown arches	***Meox1***<br><br>Homozygous nulls show hemi-vertebrae, and rib, vertebral, and cranial-vertebral fusions (103220)	Z (Dauer, Currie, & Berger, 2018)
Mesenchyme homeobox 2	***MEOX2***<br><br>GWAS AIS	***meox2a/b***<br><br>no data	***Meox2***<br><br>Homozygous nulls have mild vertebral and rib anomalies, reduced muscle mass (103219)	H (Kou et al., 2019)
Mesoderm posterior ab	***MESP2***<br><br>spondylocostal dysostosis 2 (recessive) (608681)	***mespab*** [*mesp2*]<br><br>vertebral arches malformed, somite boundaries disturbed	***Mesp2***<br><br>Homozygous nulls show absence of segmented somites, fused vertebrae and dorsal root ganglia (1096325)	Z (Yabe, Hoshijima, Yamamoto, & Takada, 2016)
Matrix metallopeptidase 14a	***MMP14***<br><br>GWAS AIS<br><br>Winchester syndrome (recessive)(277940)	***mmp14a/b***<br><br>Reduced BMD, abnormal ossification	***Mmp14/Mt1-mmp***<br><br>Homozygous nulls show craniofacial anomalies and skeletal dysplasia, reduced bone and cartilage formation, and other defects (101900)	H (Yu et al 2023)
MKS transition zone complex subunit 1	***MKS1***	***mks1***	***Mks1***	Z (J. Wang et al., 2022)

Disease Models & Mechanisms • Supplementary information

	Joubert syndrome (recessive) (66171210)<br><br>Bardet-Biedl syndrome (recessive) 615990)<br><br>Meckel syndrome 1 (recessive) (249000)	scoliosis, death	Null homozygotes shown skeletal defects and other developmental anomalies (3584243)	
Myotubularin related protein 11	***MTMR11***<br><br>GWAS AIS	***mtmr11***<br><br>no data	***Mtmr11***<br><br>(2652817)	H (Kou et al., 2019)
Myeloid-associated differentiation marker-like protein 2 like	***MYADM or MYADML***	***myadml2l***<br><br>scoliosis	***Myadm***<br><br>(1355332)	Z (Henke et al., 2017)
Myosin heavy chain 3	***MYH3***<br><br>Freeman-Sheldon, Sheldon_Hall; Contractures, pterygia, and spondylocarpostarsal fusion syndrome 1a (dominant) (193700;618436;178110);	***myh3***<br><br>congenital scoliosis, vertebral fusions & motor deficits	***Myh3***<br><br>Homozygous nulls show altered myofiber size, number and type (1339709)	Z (Henke et al., 2017)

Disease Models & Mechanisms • Supplementary information

	contractures, pterygia, and spondylocarpotarsal fusion syndrome 1b (recessive) (618469)			
NK3 homeobox 2	***NKX3.2***<br><br>spondylo-megaepiphyseal-metaphyseal dysplasia (recessive) (613330)	***nkx3.2***<br><br>Parapophyses absent, posterior skull, anterior vertebra abnormal	***Nkx3.2***<br><br>Homozygous nulls show dysplasia of the vertebral column and cranium (108015)	Z (Waldmann et al., 2021)
5'-nucleotidase domain containing 1	***NT5DC1***<br><br>GWAS AIS	***nt5dc1***<br><br>no data	***Nt5dc1***<br><br>(2442446)	H (Kou et al., 2019)
Centriole and centriolar satellite protein	***OFD1***<br><br>Joubert syndrome 1 (X-rec) (300804); Orofaciodigital syndrome I (X-dominant) (311200) Simpson-Golabi-Behmel syndrome, type 2 (X-recessive) (300209)	***ofd1***<br><br>scoliosis	***Ofd1***<br><br>Hemizygous conditional loss leads to left-right patterning defects (1350328)	Z (Xie et al., 2025~~3~~)
Pantothenate kinase 1	***PANK1***	***pank1a/ b***	***Pank1***	M (Rios et al., 2021)

		no data	Metabolic defects in homozygous null mice (1922985)<br><br>Homozygous missense allele shows spine deformity	
Pappalysin	***PAPPA***	***pappa2***<br><br>no data	***Pappa***<br><br>Homozygous nulls show decreased birth body size, reduced female fertility<br><br>(97479)<br><br>Homozygous missense allele shows spine deformity	M (Rios et al., 2021)
Paired-box transcription factor 1	***PAX1/LINC01432***<br><br>GWAS AIS<br><br>Otofaciocervical syndrome 2 with T-cell deficiency (recessive) (615560)	***pax1a***<br><br>ceratobranchial cartilage defect	***Pax1***<br><br>Homozygous nulls show congenital spine malformation; kinked tail (97485)	Z (Y. H. Liu, Lin, & Hwang, 2020)<br><br>H(Sharma et al 2015)
Piezo type mechanosensitive ion channel component 1	***PIEZO1***<br><br>Dehydrated hereditary stomatocytosis with or without pseudohyperkalemia and/or perinatal edema (dominant)	***piezo1***<br><br>bone malformation and juvenile scoliosis	***Piezo1***<br><br>(3603204)	Z (Ramli et al., 2024)

Disease Models & Mechanisms • Supplementary information

	(194380)<br><br>Lymphatic malformation 6 (recessive) (166843)			
Piezo type mechanosensitive ion channel component 2	***PIEZO2***<br><br>Arthrogryposis, distal, with impaired proprioception and touch (recessive) (617146)<br><br>Arthrogryposis, distal, type 3, type 5 (dominant) (114300, 108145)	***piezo2***<br><br>propioception	***Piezo2***<br><br>Deletion in proprioceptive neurons causes scoliosis<br><br>(1918781)	M (Blecher et al., 2017)
Polycystic kidney disease 2-like 1	***PKD2L1***	***pkd2l1***<br><br>scoliosis	***Pkd2l1***<br><br>(1352448)	Z (Marie-Hardy, Courtin, Pascal-Moussellard, Zakine, & Brice, 2023)
RNA polymerase III subunit B	***POLR3B***<br><br>Charcot-Marie-Tooth disease, demyelinating, type 1I (dominant) 619742)<br><br>Leukodystrophy, hypomyelinating, 8, with or without	***polr3b***<br><br>no data	***Polr3b***<br><br>Homozygous missense allele shows spine deformity<br><br>(1917678)	M (Rios et al., 2021)

Disease Models & Mechanisms • Supplementary information

	oligodontia and/or hypogonadotropic hypogonadism (recessive) (614381)			
Proteasome 20S subunit alpha 5	***PSMA5***	***psma5***<br><br>decreased brain size	***Psma5***<br><br>Homozygous missense allele shows spine deformity<br><br>1347009	M (Rios et al., 2021)
Protein tyrosine kinase 7a	***PTK7***	***ptk7a***<br><br>idiopathic-like scoliosis; maternal zygotic shows congenital scoliosis<br><br>***ptk7b*** no data	***Ptk7***<br><br>Homozygous nulls show neural tube defects (1918711)	Z (Hayes et al., 2014)
Plexin A2	***PLXNA2***<br><br>GWAS AIS	***plxna2***	***Plxna2***<br><br>Homozygous nulls show neuronal cell defects (107684)	H (Kou et al., 2019)
Poc5 centriolar protein	***POC5***<br><br>AIS	***poc5***<br><br>late onset idiopathic scoliosis	***Poc5***<br><br>(1914713)	H (Patten et al., 2015)
protein phosphatase 2, regulatory subunit B'', beta	***PPP2R3B***	***ppp2r3b***<br><br>scoliosis by 36 dpf, worsening with age. Reduced bone	***No direct ortholog***	Z H (Seda, Crespo, Corcelli, Osborn, & Jenkins, 2023)

Disease Models & Mechanisms • Supplementary information

		density		
RAB32a, member RAS oncogene family	***RAB32***<br><br>Susceptibility to Parkinson Disease (dominant) (620923)	***rab32a***<br><br>congenital scoliosis<br><br>notochord vacuoles	***Rab32***<br><br>(1915094)	Z (Ellis, Bagwell, & Bagnat, 2013)
RPGR interacting protein 1 like	***RPGRIP1L***<br><br>Joubert syndrome 7 (recessive) (611560)<br><br>Meckel syndrome 5 (recessive) (611561)	***rgrip1l***<br><br>multiple bend scoliosis	***Rpgrip1L***<br><br>Homozygous nulls show show exencephaly, polydactyly, laterality defects, abnormal neural tube patterning, cleft lip, micro- and anophthalmia, other developmental defects and early death<br><br>(1920563)	Z (J. Wang et al., 2022)
Signal peptide, CUB domain and EGF domain containing 3	*SCUBE3*<br><br>Short stature, facial dysmorphism, and skeletal anomalies with or without cardiac anomalies (recessive) (619184)	*scube3*<br><br>required for hedgehog signaling	*Scube3*<br><br>(3045253)<br><br>Homozygous missense allele shows spine deformity	Z (Johnson et al., 2012)<br><br>M (Rios et al., 2021)
Solute carrier family 6 member 9	***SLC6A9***	***slc6A9***	***Slc6a9***	Z H (X. Wang et al., 2024)

Disease Models & Mechanisms • Supplementary information

	AIS<br><br>Glycine encephalopathy with normal serum glycine (recessive) (617301)	scoliosis	Neonatal lethal in homozygous nulls (95760)	
Solute carrier family 39 member 8	***SLC39A8***<br><br>AIS<br><br>Congenital disorder of glycosylation, type IIn (recessive) (616721)	***slc39a8***<br><br>scoliosis	***Slc39a8***<br><br>Embryonic lethal in homozygous nulls (1914797)	Z H (Haller et al., 2018)
SRY-box transcription factor 6	***SOX6***<br><br>Tolchin-Le Caignec syndrome (dominant) (618971)	***sox6***<br><br>failure to thrive, rare scoliosis	***Sox6***<br><br>Homozygous nulls show cardiac defects and early postnatal lethality (98368)	Z (Jackson et al., 2015)<br><br>H (Wise CA, 2020)
SRY-box transcription factor 9a	***SOX9***<br><br>AIS GWAS<br><br>46XX sex reversal 2, 46XY sex reversal 10 (dominant) (278850, 616425)<br><br>Acampomelic	***sox9a sox9b***<br><br>endochondral bone defects (jaw)	***Sox9***<br><br>Heterozygous nulls show perinatal lethality, cleft palate, hypoplasia of cartilage-derived skeletal structures, and premature mineralization in many bones (98371	Z (Yan et al., 2002)<br><br>H (Miyake et al., 2013)

Disease Models & Mechanisms • Supplementary information

	campomelic dysplasia, campomelic dysplasia, campomelic dysplasia with autosomal sex reversal (dominant) 114290)			
SCO-spondin	***SSPOP (pseudogene)***	***sspo***<br><br>scoliosis	***Spop***<br><br>(1343085)	Z (Rose et al., 2020; Troutwine et al., 2020)
Signal transducer and activator of transcription 3	***STAT3***<br><br>HyperIgE syndrome (dominant) (615952, 147060)	***stat3***<br><br>Scoliosis, inflammation, failure to thrive	***Stat3***<br><br>Null homozygotes show early embryonic lethality<br><br>(103038)	Z (Y. Liu, Sepich, & Solnica-Krezel, 2017)
T-box transcription factor 1	***TBX1***<br><br>GWAS AIS<br><br>Conotruncal anomaly face syndrome (dominant) (217095)<br><br>DiGeorge Syndrome (dominant)	***tbx1***<br><br>abnormal jaw cartilage	***Tbx1***<br><br>Null homozygotes show neonatal lethality and various developmental defects<br><br>(98493)	H (Kou et al., 2019)

Disease Models & Mechanisms • Supplementary information

	(188400)<br><br>Tetalogy of Fallot (dominant) (187500)<br><br>Velocardiofacial syndrome (dominant)(192430)			
T-box transcription factor 6	***TBX6***<br><br>Spondylocostal dysostosis 5 (122600)<br><br>Susceptibility to congenital scoliosis	***tbx6***<br><br>tail truncation, segmentation	***Tbx6***<br><br>Homozygous null die in early organogenesis with defects in somite development, neural tube development, and laterality defects (102539)	Z (Lleras-Forero et al., 2020)<br><br>H (Wu et al., 2015)
UDP-glucuronate decarboxylase 1	***UNCX***<br><br>GWAS AIS	***uncx***<br><br>no data	***Uncx***<br><br>Homozygous nulls show severe skeletal defects, including absence of spine pedicles, transverse processes and proximal ribs (108013)	H (Yonezawa et al., 2023)
Urotensin-related peptide 1	***UTS2B***	***urp1***<br><br>scoliosis	***Uts2b***<br><br>(2677064)	Z (Bearce et al., 2022) (Gaillard et al., 2023)
Urotensin-related peptide 2	***UTS2***	***urp2***<br><br>scoliosis	***Uts2***<br><br>(1346329)	Z (Bearce et al., 2022b) (Gaillard et al., 2023)
UDP-glucuronic acid	***UXS1***	***uxs1***	***Uxs1***	M (Rios et al., 2021)

Disease Models & Mechanisms • Supplementary information

decarboxylase 1		jaw cartilage	(1915133)<br><br>Homozygous missense allele shows spine deformity	
Urotensin-2 receptor 3	***UTS2R***<br><br>Single variant associated with AIS	***uts2r3***<br><br>scoliosis	***Uts2r***<br><br>(2183450)	Z (Bearce et al., 2022; Gaillard et al., 2023)
VAC14 component of PIKFYVE complex	***VAC14***<br><br>Striatonigral degeneration, childhood-onset (recessive) (617054)	***vac14***<br><br>no data	***vac14***<br><br>Homozygous nulls show display early postnatal lethality and lesions in multiple regions of the brain (2157980)<br><br>Homozygous missense allele shows spine deformity	M (Rios et al., 2021)
VANGL planar cell polarity protein 2	***VANGL2***<br><br>Neural tube defects (dominant) (182940)<br><br>Congenital scoliosis	***vangl2***<br><br>Congenital scoliosis	***Vangl2***<br><br>Homozygous nulls are neonatal lethal; developmental defects are seen in nerve, skeletal, vestibular, and respiratory systems; heterozygotes show looped tails (2135272)	Z (M. Wang et al., 2024)<br><br>H (Feng et al., 2024)
Wingless-type MMTV integration site family, member 3A	***WNT3A***	***wnt3a***<br><br>caudal scoliosis, notochord vs. neural fate	***Wnt3a***<br><br>Homozygous nulls are early embryonic lethal with failure of somitogenesis and notochord	H (Kou et al., 2019)

Disease Models & Mechanisms • Supplementary information

			development; homozygotes for a hypomorphic allele show vertebral defects loss of caudal vertebrae (98956)	
Zinc finger, MYND-type containing 1***0***	***ZMYND10***<br><br>AIS<br>Ciliary dyskinesia, primary, 22 (recessive) (615444)	***zmynd10***<br><br>scoliosis	***Zmynd10***<br><br>Homozygous nulls show postnatal growth retardation, hydrocephalus, situs inversus, of ciliary motility (2387863)	Z, H (Y. Wang et al., 2020)

Abbreviations: AIS,adolescent idiopathic scoliosis; BMD, bone mineral density; cKO, conditional knockout; CNS, central nervous system; GWAS, genome-wide association study; IVD, intervertebral disc; KO, knockout; LOF, loss-of-function; OE, overexpression; TMD, tissue mineral density

Disease Models & Mechanisms • Supplementary information

Table 1 References

Apschner, A., Huitema, L. F., Ponsioen, B., Peterson-Maduro, J. and Schulte-Merker, S. (2014). Zebrafish enpp1 mutants exhibit pathological mineralization, mimicking features of generalized arterial calcification of infancy (GACI) and pseudoxanthoma elasticum (PXE). *Dis Model Mech* **7**, 811-22.

Bagwell, J., Norman, J., Ellis, K., Peskin, B., Hwang, J., Ge, X., Nguyen, S. V., McMenamin, S. K., Stainier, D. Y. and Bagnat, M. (2020). Notochord vacuoles absorb compressive bone growth during zebrafish spine formation. *Elife* **9**.

Bearce, E. A., Irons, Z. H., O'Hara-Smith, J. R., Kuhns, C. J., Fisher, S. I., Crow, W. E. and Grimes, D. T. (2022). Urotensin II-related peptides, Urp1 and Urp2, control zebrafish spine morphology. *Elife* **11**.

Blackwell, D. L., Fraser, S. D., Caluseriu, O., Vivori, C., Tyndall, A. V., Lamont, R. E., Parboosingh, J. S., Innes, A. M., Bernier, F. P. and Childs, S. J. (2022). Hnrnpul1 controls transcription, splicing, and modulates skeletal and limb development in vivo. *G3 (Bethesda)* **12**.

Blecher, R., Krief, S., Galili, T., Biton, I. E., Stern, T., Assaraf, E., Levanon, D., Appel, E., Anekstein, Y., Agar, G. et al. (2017). The Proprioceptive System Masterminds Spinal Alignment: Insight into the Mechanism of Scoliosis. *Dev Cell* **42**, 388-399 e3.

Chae, U., Park, N. R., Kim, E. S., Choi, J. Y., Yim, M., Lee, H. S., Lee, S. R., Lee, S., Park, J. W. and Lee, D. S. (2018). IDH2-deficient mice develop spinal deformities with aging. *Physiol Res* **67**, 487-494.

Cooper, W. J., Wirgau, R. M., Sweet, E. M. and Albertson, R. C. (2013). Deficiency of zebrafish fgf20a results in aberrant skull remodeling that mimics both human cranial disease and evolutionarily important fish skull morphologies. *Evol Dev* **15**, 426-41.

Dauer, M. V. P., Currie, P. D. and Berger, J. (2018). Skeletal malformations of Meox1-deficient zebrafish resemble human Klippel-Feil syndrome. *J Anat* **233**, 687-695.

Ellis, K., Bagwell, J. and Bagnat, M. (2013). Notochord vacuoles are lysosome-related organelles that function in axis and spine morphogenesis. *J Cell Biol* **200**, 667-79.

Feng, X., Ye, Y., Zhang, J., Zhang, Y., Zhao, S., Mak, J. C. W., Otomo, N., Zhao, Z., Niu, Y., Yonezawa, Y. et al. (2024). Core planar cell polarity genes VANGL1 and VANGL2 in predisposition to congenital vertebral malformations. *Proc Natl Acad Sci U S A* **121**, e2310283121.

Disease Models & Mechanisms • Supplementary information

Fradet, A. and Fitzgerald, J. (2017). INPPL1 gene mutations in opsismodysplasia. *J Hum Genet* **62**, 135-140.

Gaillard, A. L., Mohamad, T., Quan, F. B., de Cian, A., Mosimann, C., Tostivint, H. and Pezeron, G. (2023). Urp1 and Urp2 act redundantly to maintain spine shape in zebrafish larvae. *Dev Biol* **496**, 36-51.

Gao, W., Chen, C., Zhou, T., Yang, S., Gao, B., Zhou, H., Lian, C., Wu, Z., Qiu, X., Yang, X. et al. (2017). Rare coding variants in MAPK7 predispose to adolescent idiopathic scoliosis. *Hum Mutat* **38**, 1500-1510.

Gistelinck, C., Kwon, R. Y., Malfait, F., Symoens, S., Harris, M. P., Henke, K., Hawkins, M. B., Fisher, S., Sips, P., Guillemyn, B. et al. (2018). Zebrafish type I collagen mutants faithfully recapitulate human type I collagenopathies. *Proc Natl Acad Sci U S A* **115**, E8037-E8046.

Gray, R. S., Gonzalez, R., Ackerman, S. D., Minowa, R., Griest, J. F., Bayrak, M. N., Troutwine, B., Canter, S., Monk, K. R., Sepich, D. S. et al. (2021). Postembryonic screen for mutations affecting spine development in zebrafish. *Dev Biol* **471**, 18-33.

Gray, R. S., Wilm, T. P., Smith, J., Bagnat, M., Dale, R. M., Topczewski, J., Johnson, S. L. and Solnica-Krezel, L. (2014). Loss of col8a1a function during zebrafish embryogenesis results in congenital vertebral malformations. *Dev Biol* **386**, 72-85.

Haller, G., Alvarado, D., McCall, K., Yang, P., Cruchaga, C., Harms, M., Goate, A., Willing, M., Morcuende, J. A., Baschal, E. et al. (2016). A polygenic burden of rare variants across extracellular matrix genes among individuals with adolescent idiopathic scoliosis. *Human Molecular Genetics* **25**, 202-9.

Haller, G., McCall, K., Jenkitkasemwong, S., Sadler, B., Antunes, L., Nikolov, M., Whittle, J., Upshaw, Z., Shin, J., Baschal, E. et al. (2018). A missense variant in SLC39A8 is associated with severe idiopathic scoliosis. *Nat Commun* **9**, 4171.

Hayes, M., Gao, X., Yu, L. X., Paria, N., Henkelman, R. M., Wise, C. A. and Ciruna, B. (2014). ptk7 mutant zebrafish models of congenital and idiopathic scoliosis implicate dysregulated Wnt signalling in disease. *Nat Commun* **5**, 4777.

Henke, K., Daane, J. M., Hawkins, M. B., Dooley, C. M., Busch-Nentwich, E. M., Stemple, D. L. and Harris, M. P. (2017). Genetic Screen for Postembryonic Development in the Zebrafish (Danio rerio): Dominant Mutations Affecting Adult Form. *Genetics* **207**, 609-623.

Jackson, H. E., Ono, Y., Wang, X., Elworthy, S., Cunliffe, V. T. and Ingham, P. W. (2015). The role of Sox6 in zebrafish muscle fiber type specification. *Skelet Muscle* **5**, 2.

Jaffe, K. M., Grimes, D. T., Schottenfeld-Roames, J., Werner, M. E., Ku, T. S., Kim, S. K., Pelliccia, J. L., Morante, N. F., Mitchell, B. J. and Burdine, R. D. (2016). c21orf59/kurly Controls Both Cilia Motility and Polarization. *Cell Rep* **14**, 1841-9.

Johnson, J. L., Hall, T. E., Dyson, J. M., Sonntag, C., Ayers, K., Berger, S., Gautier, P., Mitchell, C., Hollway, G. E. and Currie, P. D. (2012). Scube activity is necessary for Hedgehog signal transduction in vivo. *Dev Biol* **368**, 193-202.

Juarez-Morales, J. L., Weierud, F., England, S. J., Demby, C., Santos, N., Grieb, G., Mazan, S. and Lewis, K. E. (2021). Evolution of lbx spinal cord expression and function. *Evol Dev* **23**, 404-422.

Karner, C. M., Long, F., Solnica-Krezel, L., Monk, K. R. and Gray, R. S. (2015). Gpr126/Adgrg6 deletion in cartilage models idiopathic scoliosis and pectus excavatum in mice. *Human Molecular Genetics* **24**, 4365-73.

Khanshour, A. M., Kou, I., Fan, Y., Einarsdottir, E., Makki, N., Kidane, Y. H., Kere, J., Grauers, A., Johnson, T. A., Paria, N. et al. (2018). Genome-wide meta-analysis and replication studies in multiple ethnicities identify novel adolescent idiopathic scoliosis susceptibility loci. *Human Molecular Genetics* **27**, 3986-3998.

Kou, I., Otomo, N., Takeda, K., Momozawa, Y., Lu, H. F., Kubo, M., Kamatani, Y., Ogura, Y., Takahashi, Y., Nakajima, M. et al. (2019). Genome-wide association study identifies 14 previously unreported susceptibility loci for adolescent idiopathic scoliosis in Japanese. *Nat Commun* **10**, 3685.

Kou, I., Takahashi, Y., Johnson, T. A., Takahashi, A., Guo, L., Dai, J., Qiu, X., Sharma, S., Takimoto, A., Ogura, Y. et al. (2013). Genetic variants in GPR126 are associated with adolescent idiopathic scoliosis. *Nat Genet* **45**, 676-9.

Li, L., Li, J., Ou, Y., Wu, J., Li, H., Wang, X., Tang, L., Dai, X., Yang, C., Wei, Z. et al. (2023). Ccdc57 is required for straightening the body axis by regulating ciliary motility in the brain ventricle of zebrafish. *J Genet Genomics* **50**, 253-263.

Li, Y., Lacerda, D. A., Warman, M. L., Beier, D. R., Yoshioka, H., Ninomiya, Y., Oxford, J. T., Morris, N. P., Andrikopoulos, K., Ramirez, F. et al. (1995). A fibrillar collagen gene, Col11a1, is essential for skeletal morphogenesis. *Cell* **80**, 423-30.

Lindstrand, A., Frangakis, S., Carvalho, C. M., Richardson, E. B., McFadden, K. A., Willer, J. R., Pehlivan, D., Liu, P., Pediaditakis, I. L., Sabo, A. et al. (2016). Copy-Number Variation Contributes to the Mutational Load of Bardet-Biedl Syndrome. *Am J Hum Genet* **99**, 318-36.

Liu, Y., Sepich, D. S. and Solnica-Krezel, L. (2017). Stat3/Cdc25a-dependent cell proliferation promotes embryonic axis extension during zebrafish gastrulation. *PLoS Genet* **13**, e1006564.

Liu, Y. H., Lin, T. C. and Hwang, S. L. (2020). Zebrafish Pax1a and Pax1b are required for pharyngeal pouch morphogenesis and ceratobranchial cartilage development. *Mech Dev* **161**, 103598.

Disease Models & Mechanisms • Supplementary information

Lleras-Forero, L., Winkler, C. and Schulte-Merker, S. (2020). Zebrafish and medaka as models for biomedical research of bone diseases. *Dev Biol* **457**, 191-205.

Marie-Hardy, L., Courtin, T., Pascal-Moussellard, H., Zakine, S. and Brice, A. (2023). The Whole-Exome Sequencing of a Cohort of 19 Families with Adolescent Idiopathic Scoliosis (AIS): Candidate Pathways. *Genes (Basel)* **14**.

McCallum-Loudeac, J., Moody, E., Williams, J., Johnstone, G., Sircombe, K. J., Clarkson, A. N. and Wilson, M. J. (2024). Deletion of a conserved genomic region associated with adolescent idiopathic scoliosis leads to vertebral rotation in mice. *Human Molecular Genetics* **33**, 787-801.

Miyake, A., Kou, I., Takahashi, Y., Johnson, T. A., Ogura, Y., Dai, J., Qiu, X., Takahashi, A., Jiang, H., Yan, H. et al. (2013). Identification of a susceptibility locus for severe adolescent idiopathic scoliosis on chromosome 17q24.3. *PLoS One* **8**, e72802.

Nitschke, Y. and Rutsch, F. (2012). Generalized arterial calcification of infancy and pseudoxanthoma elasticum: two sides of the same coin. *Front Genet* **3**, 302.

Ogura, Y., Kou, I., Miura, S., Takahashi, A., Xu, L., Takeda, K., Takahashi, Y., Kono, K., Kawakami, N., Uno, K. et al. (2015). A Functional SNP in BNC2 Is Associated with Adolescent Idiopathic Scoliosis. *Am J Hum Genet* **97**, 337-42.

Osborn, D. P., Roccasecca, R. M., McMurray, F., Hernandez-Hernandez, V., Mukherjee, S., Barroso, I., Stemple, D., Cox, R., Beales, P. L. and Christou-Savina, S. (2014). Loss of FTO antagonises Wnt signaling and leads to developmental defects associated with ciliopathies. *PLoS One* **9**, e87662.

Pang, L., Zhang, Z., Shen, Y., Cheng, Z., Gao, X., Zhang, B., Wang, X. and Tian, H. (2020). Mutant dlx3b disturbs normal tooth mineralization and bone formation in zebrafish. *PeerJ* **8**, e8515.

Patten, S. A., Margaritte-Jeannin, P., Bernard, J. C., Alix, E., Labalme, A., Besson, A., Girard, S. L., Fendri, K., Fraisse, N., Biot, B. et al. (2015). Functional variants of POC5 identified in patients with idiopathic scoliosis. *J Clin Invest* **125**, 1124-8.

Pontoizeau, C., Gaborit, C., Tual, N., Simon-Sola, M., Rotaru, I., Benoist, M., Colella, P., Lamaziere, A., Brassier, A., Arnoux, J. B. et al. (2024). Successful treatment of severe MSUD in Bckdhb(-/-) mice with neonatal AAV gene therapy. *J Inherit Metab Dis* **47**, 41-49.

Pottie, L., Adamo, C. S., Beyens, A., Lutke, S., Tapaneeyaphan, P., De Clercq, A., Salmon, P. L., De Rycke, R., Gezdirici, A., Gulec, E. Y. et al. (2021). Bi-allelic premature truncating variants in LTBP1 cause cutis laxa syndrome. *Am J Hum Genet* **108**, 2386-2388.

Raman, R., Antony, M., Nivelle, R., Lavergne, A., Zappia, J., Guerrero-Limon, G., Caetano da Silva, C., Kumari, P., Sojan, J. M., Degueldre, C. et al. (2024). The Osteoblast Transcriptome in Developing Zebrafish Reveals Key Roles for Extracellular Matrix Proteins Col10a1a and Fbln1 in Skeletal Development and Homeostasis. *Biomolecules* **14**.

Ramli, Aramaki, T., Watanabe, M. and Kondo, S. (2023). Piezo1 mutant zebrafish as a model of idiopathic scoliosis. *Front Genet* **14**, 1321379.

Rebello, D., Wohler, E., Erfani, V., Li, G., Aguilera, A. N., Santiago-Cornier, A., Zhao, S., Hwang, S. W., Steiner, R. D., Zhang, T. J. et al. (2023). COL11A2 as a candidate gene for vertebral malformations and congenital scoliosis. *Human Molecular Genetics* **32**, 2913-2928.

Rios, J. J., Denton, K., Yu, H., Manickam, K., Garner, S., Russell, J., Ludwig, S., Rosenfeld, J. A., Liu, P., Munch, J. et al. (2021). Saturation mutagenesis defines novel mouse models of severe spine deformity. *Dis Model Mech*.

Rivero, O., Sich, S., Popp, S., Schmitt, A., Franke, B. and Lesch, K. P. (2013). Impact of the ADHD-susceptibility gene CDH13 on development and function of brain networks. *Eur Neuropsychopharmacol* **23**, 492-507.

Rose, C. D., Pompili, D., Henke, K., Van Gennip, J. L. M., Meyer-Miner, A., Rana, R., Gobron, S., Harris, M. P., Nitz, M. and Ciruna, B. (2020). SCO-Spondin Defects and Neuroinflammation Are Conserved Mechanisms Driving Spinal Deformity across Genetic Models of Idiopathic Scoliosis. *Curr Biol* **30**, 2363-2373 e6.

Seda, M., Crespo, B., Corcelli, M., Osborn, D. P. and Jenkins, D. (2023). A CRISPR/Cas9-generated mutation in the zebrafish orthologue of PPP2R3B causes idiopathic scoliosis. *Sci Rep* **13**, 6783.

Shapiro, F. (2016). Disordered vertebral and rib morphology in pudgy mice. Structural relationships to human scoliosis. *Adv Anat Embryol Cell Biol* **221**, 1-123.

Sharma, S., Londono, D., Eckalbar, W. L., Gao, X., Zhang, D., Mauldin, K., Kou, I., Takahashi, A., Matsumoto, M., Kamiya, N. et al. (2015). A PAX1 enhancer locus is associated with susceptibility to idiopathic scoliosis in females. *Nat Commun* **6**, 6452.

Song, P., Fogerty, J., Cianciolo, L. T., Stupay, R. and Perkins, B. D. (2020). Cone Photoreceptor Degeneration and Neuroinflammation in the Zebrafish Bardet-Biedl Syndrome 2 (bbs2) Mutant Does Not Lead to Retinal Regeneration. *Front Cell Dev Biol* **8**, 578528.

Takahashi, Y., Kou, I., Takahashi, A., Johnson, T. A., Kono, K., Kawakami, N., Uno, K., Ito, M., Minami, S., Yanagida, H. et al. (2011). A genome-wide association study identifies common variants near LBX1 associated with adolescent idiopathic scoliosis. *Nat Genet* **43**, 1237-40.

Terhune, E. A., Cuevas, M. T., Monley, A. M., Wethey, C. I., Chen, X., Cattell, M. V., Bayrak, M. N., Bland, M. R., Sutphin, B., Trahan, G. D. et al. (2021). Mutations in KIF7 implicated in idiopathic scoliosis in humans and axial curvatures in zebrafish. *Hum Mutat* **42**, 392-407.

Troutwine, B. R., Gontarz, P., Konjikusic, M. J., Minowa, R., Monstad-Rios, A., Sepich, D. S., Kwon, R. Y., Solnica-Krezel, L. and Gray, R. S. (2020). The Reissner Fiber Is Highly Dynamic In Vivo and Controls Morphogenesis of the Spine. *Curr Biol* **30**, 2353-2362 e3.

Van De Weghe, J. C., Rusterholz, T. D. S., Latour, B., Grout, M. E., Aldinger, K. A., Shaheen, R., Dempsey, J. C., Maddirevula, S., Cheng, Y. H., Phelps, I. G. et al. (2017). Mutations in ARMC9, which Encodes a Basal Body Protein, Cause Joubert Syndrome in Humans and Ciliopathy Phenotypes in Zebrafish. *Am J Hum Genet* **101**, 23-36.

Van Gils, M., Willaert, A., De Vilder, E. Y. G., Coucke, P. J. and Vanakker, O. M. (2018). Generation and Validation of a Complete Knockout Model of abcc6a in Zebrafish. *J Invest Dermatol* **138**, 2333-2342.

Waldmann, L., Leyhr, J., Zhang, H., Ohman-Magi, C., Allalou, A. and Haitina, T. (2021). The broad role of Nkx3.2 in the development of the zebrafish axial skeleton. *PLoS One* **16**, e0255953.

Wang, J., Thomas, H. R., Thompson, R. G., Waldrep, S. C., Fogerty, J., Song, P., Li, Z., Ma, Y., Santra, P., Hoover, J. D. et al. (2022). Variable phenotypes and penetrance between and within different zebrafish ciliary transition zone mutants. *Dis Model Mech* **15**.

Wang, M., Zhao, S., Shi, C., Guyot, M. C., Liao, M., Tauer, J. T., Willie, B. M., Cobetto, N., Aubin, C. E., Kuster-Schock, E. et al. (2024a). Planar cell polarity zebrafish models of congenital scoliosis reveal underlying defects in notochord morphogenesis. *Development* **151**.

Wang, X., Yue, M., Cheung, J. P. Y., Cheung, P. W. H., Fan, Y., Wu, M., Wang, X., Zhao, S., Khanshour, A. M., Rios, J. J. et al. (2024b). Impaired glycine neurotransmission causes adolescent idiopathic scoliosis. *J Clin Invest* **134**.

Disease Models & Mechanisms • Supplementary information

Wang, Y., Liu, Z., Yang, G., Gao, Q., Xiao, L., Li, J., Guo, C., Troutwine, B. R., Gray, R. S., Xie, L. et al. (2020). Coding Variants Coupled With Rapid Modeling in Zebrafish Implicate Dynein Genes, dnaaf1 and zmynd10, as Adolescent Idiopathic Scoliosis Candidate Genes. *Front Cell Dev Biol* **8**, 582255.

Wise CA, S. D., Ushiki A, Khanshour A, Kidane YH, Makki N, Gurnett CA, Gray RS, Rios JJ, Ahituv N, Solnica-Krezel L. (2020). The cartilage matrisome in adolescent idiopathic scoliosis. *Bone Research* **8**.

Wu, N., Ming, X., Xiao, J., Wu, Z., Chen, X., Shinawi, M., Shen, Y., Yu, G., Liu, J., Xie, H. et al. (2015). TBX6 null variants and a common hypomorphic allele in congenital scoliosis. *New England Journal of Medicine* **372**, 341-50.

Xie, Y., Liu, Z., Li, Q., Li, T., Guo, J., Hu, M., Sun, J., Jiang, H., Wang, J., Wang, S. et al. (2025). Myeloma interaction with bone marrow stromal cells suppresses ciliogenesis and osteogenic potential in myeloma bone disease. *Sci Transl Med* **17**, eadq2961.

Yabe, T., Hoshijima, K., Yamamoto, T. and Takada, S. (2016). Quadruple zebrafish mutant reveals different roles of Mesp genes in somite segmentation between mouse and zebrafish. *Development* **143**, 2842-52.

Yamauchi, H., Goto, M., Katayama, M., Miyake, A. and Itoh, N. (2011). Fgf20b is required for the ectomesenchymal fate establishment of cranial neural crest cells in zebrafish. *Biochem Biophys Res Commun* **409**, 705-10.

Yan, Y. L., Miller, C. T., Nissen, R. M., Singer, A., Liu, D., Kirn, A., Draper, B., Willoughby, J., Morcos, P. A., Amsterdam, A. et al. (2002). A zebrafish sox9 gene required for cartilage morphogenesis. *Development* **129**, 5065-79.

Yonezawa, Y., Guo, L., Kakinuma, H., Otomo, N., Yoshino, S., Takeda, K., Nakajima, M., Shiraki, T., Ogura, Y., Takahashi, Y. et al. (2023). Identification of a Functional Susceptibility Variant for Adolescent Idiopathic Scoliosis that Upregulates Early Growth Response 1 (EGR1)-Mediated UNCX Expression. *J Bone Miner Res* **38**, 144-153.

Yu, H., Khanshour, A. M., Ushiki, A., Otomo, N., Koike, Y., Einarsdottir, E., Fan, Y., Antunes, L., Kidane, Y. H., Cornelia, R. et al. (2024). Association of genetic variation in COL11A1 with adolescent idiopathic scoliosis. *Elife* **12**.

Zhao, N., Han, D., Liu, Y., Li, Y., Zeng, L., Wang, Y. and Feng, H. (2016). DLX3 negatively regulates osteoclastic differentiation through microRNA-124. *Exp Cell Res* **341**, 166-76.
